# Contrasting patterns of microbial dominance in the *Arabidopsis thaliana* phyllosphere

**DOI:** 10.1073/pnas.2211881119

**Published:** 2022-12-20

**Authors:** Derek S. Lundberg, Roger de Pedro Jové, Pratchaya Pramoj Na Ayutthaya, Talia L. Karasov, Or Shalev, Karin Poersch, Wei Ding, Anita Bollmann-Giolai, Ilja Bezrukov, Detlef Weigel

**Affiliations:** ^a^Department of Molecular Biology, Max Planck Institute for Biology Tübingen, Tübingen 72076, Germany; ^b^Swedish University of Agricultural Sciences, Uppsala 75651, Sweden

**Keywords:** S​phi​ngo​mon​as, Pseudomonas, phyllosphere, genomics, interactions

## Abstract

*Sphingomonas* is one of the most abundant bacterial genera in the phyllosphere of wild *Arabidopsis thaliana*, but relative to *Pseudomonas*, the ecology of *Sphingomonas* and its interaction with plants is poorly described. We analyzed the genomic features of over 400 *Sphingomonas* isolates collected from local *A. thaliana* populations, which revealed much higher intergenomic diversity than for the considerably more uniform *Pseudomonas* isolates found in the same host populations. Variation in *Sphingomonas* plasmid complements and additional genomic features suggest high adaptability of this genus, and the widespread presence of protein secretion systems hints at frequent biotic interactions. While some of the isolates showed plant-protective phenotypes in lab tests, this was a rare trait. To begin to understand the extent of strain sharing across alternate hosts, we employed amplicon sequencing and a bulk-culturing metagenomics approach on both *A. thaliana* and neighboring plants. Our data reveal that both *Sphingomonas* and *Pseudomonas* thrive on other diverse plant hosts, but that *Sphingomonas* is a poor competitor in dying or dead leaves.

## Significance

Although *Sphingomonas* is often the most abundant bacterial taxon on many plant species, plant-associated members of this genus have not been studied in a comprehensive and ecologically rooted manner. Through sequencing hundreds of *Sphingomonas* genomes from *Arabidopsis thaliana* and other plants, 16S rRNA gene amplicon surveys, bulk metagenomes of cultured *Sphingomonas*, and comparisons and competition studies with local *Pseudomonas*, we show that *Sphingomonas* on wild plants establish consistently abundant and diverse populations that may include pathogen-suppressive members. Their success in the phyllosphere may depend on occupying different niches than *Pseudomonas*, or on a functioning plant immune system, as they are quickly outcompeted by *Pseudomonas* when forced into the same spatial location in macerated leaves.

Most ecosystems, including host-associated microbiomes, are composed of a handful of common species and a wide assortment of rarer species (1, [Bibr r2], 3). Common species are frequently implicated in direct interactions with the host. For example in the human gut, the genera *Bacteroides* and *Prevotella*, which often occupy 20% or more of the entire community (4), modulate the host immune system (5). Similarly, on and inside plant leaves, the Proteobacterial genera *Sphingomonas* and *Pseudomonas* are among the most common bacterial taxa, not only on the model plant *Arabidopsis thaliana* (6–10), but also on many other species across continents (11–18).

*Pseudomonas*–leaf interactions are widely studied (13, 19–21), largely due to agricultural plant pathogens in the genus (13). *Sphingomonas*–leaf interactions, however, are not well understood at a genetic level or population level, despite the ubiquity of *Sphingomonas* on plant leaves and increasing reports of plant beneficial members of this genus (22–24). *Sphingomonas* derives its name from its membrane-bound sphingolipids (25), structural and signaling molecules that are common in eukaryotes but found only in a few bacterial taxa (4, 26). These taxa include the previously mentioned *Bacteroides* and *Prevotella* of the gut, whose sphingolipids interact with the mammalian host and even influence host nutrition (4, 27). *Sphingomonas* also associate with plant roots (23, 28, 29) and seeds (22) and are common in soil and freshwater (30) among other habitats. Some strains can improve plant growth and abiotic stress tolerance in contaminated soils (31), and various others can promote plant growth through the production of growth regulators (32). *Sphingomonas* strains affect the abundances of other microbes (6, 22, 33, 34), and some protect against pathogenic bacteria (22, 35) or fungi (23).

In our previous studies of microbes colonizing local *A. thaliana* in southwest (SW) Germany, we also initially focused on characterizing *Pseudomonas* populations, and we use those results here as a benchmark for understanding *Sphingomonas* ecology. We reported that *Pseudomonas* varied widely in bacterial load on individual plants, ranging from being nearly absent to very high titers (8, 10, 19). At the genomic level, 1,524 *Pseudomonas* isolates from local *A. thaliana* plants consisted primarily of a lineage of closely related *P*seudomonas* viridiflava* (“OTU5” in the original publication and hereafter referred to as Pv-ATUE5) (36) that shared at least 99.9% nucleotide identity in their core genomes and the same diagnostic partial 16S rRNA gene sequence (19). Despite their similarity to each other, Pv-ATUE5 strains differed widely in pathogenicity in a gnotobiotic system, and phylogenetic analysis suggested that subgroups of Pv-ATUE5 diverged around 300,000 y ago, consistent with complex selective pressures that have not favored conquest by a single isolate (19).

In this work, we sought to characterize local *Sphingomonas* populations at the strain level to ask if, like Pv-ATUE5, a single lineage rules the local *A. thaliana* bacterial community, and to determine general genetic features of plant-associated *Sphingomonas*. We also extended our survey onto neighboring individuals of various plant species at the site and asked to what extent *Sphingomonas* and *Pseudomonas* strains common to *A. thaliana* are generalists, and thus face selective pressures shaped by life on multiple host plants. This work reinforces the notion that individual *Sphingomonas* strains likely have broad host ranges and that they have genetic features equipping them for a multitude of biotic interactions, suggesting major roles not only in the assembly of leaf communities but also in influencing the general means by which plants sense and respond to nonpathogenic or beneficial microbes.

## Results

### *Sphingomonas* Colonizes *A. thaliana* More Consistently than *Pseudomonas*.

**Fig. 1.**
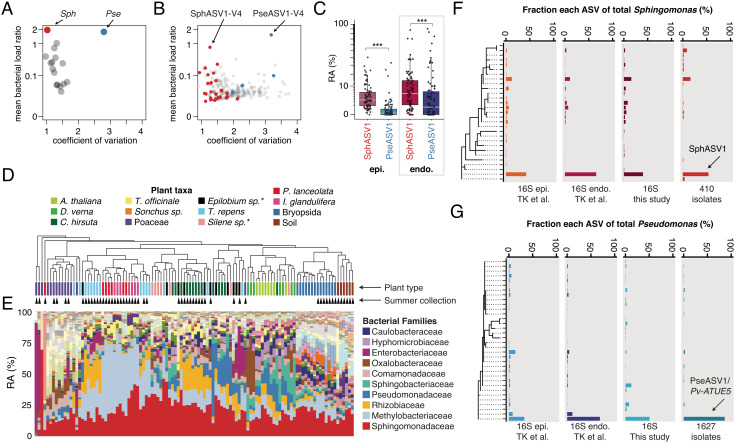
*Sphingomonas* and *Pseudomonas* are abundant and culturable, but differ in consistency of colonization. (*A*) *Sphingomonas* (red, Sph) has a similarly high bacterial load to *Pseudomonas* (blue, Pse) across 176 *A. thaliana* plants from (10), but among the lowest coefficients of variation (SD / mean abundance) of other major genera (gray). (*B*) Similar to (*A*), but showing the V4 16S rDNA sequences that make up the most abundant genera. *Sphingomonas* ASVs have on average low coefficients of variation, while *Pseudomonas* ASVs have on average higher coefficients of variation. (*C*) The median relative abundance (RA) of SphASV1 is higher than PseASV1 both epiphytically (epi.) and endophytically (endo.) (***, *P* < 0.001, FDR-adjusted Mann–Whitney *U* test). Boxes enclose the interquartile range (IQR) with whiskers extending to up to 1.5 times the IQR. (*D*) Plant samples in the present study clustered by the Bray–Curtis dissimilarity of the relative abundance of bacterial families. Summer collection indicated with black triangles. A "*" represents uncertainty in the plant taxon name. (*E*) Stacked bars represent the relative abundances (RA) of bacterial families and correspond to the plant samples directly above in *D* The colors identifying the top 10 bacterial families are shown in the legend. (*F*) Relative abundances of *Sphingomonas* ASVs detected epiphytically (epi.) and endophytically (endo.) in (19) ("TK et al."), this study, and in 410 locally cultured isolates from multiple plant species. SphASV1 is indicated. (*G*) Same as in *F*, but for *Pseudomonas*, with 1,627 isolates combining 1,524 isolates from (19) and 103 additional isolates from this study.

We first analyzed a previous microbiome dataset from wild *A. thaliana* rosettes, for which we had used plant DNA as a scaling factor to calculate the bacterial load of each amplicon sequence variant (ASV) in the V4 hypervariable region of the 16S rRNA gene (rDNA) (10). After classifying ASVs at the genus level, *Sphingomonas* and *Pseudomonas* were approximately equal in bacterial load overall, and twice as abundant as the third most common genus (*Pedobacter*). However, while *Pseudomonas* loads varied widely between samples, *Sphingomonas* was among the most consistent genera (Fig. 1*A*). Impressively, the consistency of *Sphingomonas* colonization also extended to individual *Sphingomonas* ASVs that independently colonized most plants (Fig. 1*B*). We named the most abundant *Sphingomonas* and *Pseudomonas* ASVs “SphASV1-V4” and “PseASV1-V4”, respectively.

### Dominant *Sphingomonas* and *Pseudomonas* ASVs Are Enriched Endophytically.

We next reanalyzed slightly longer rDNA sequences in the V3V4 hypervariable regions from a different set of wild *A. thaliana* rosettes in ref. 19, for which we had partitioned *A. thaliana* leaf epiphytes from endophytes. We found a single V3V4 ASV each that matched SphASV1-V4 and PseASV1-V4, and we refer to these longer sequences henceforth simply as SphASV1 and PseASV1. PseASV1 exactly matched the representative sequence previously used to define the abundant Pv-ATUE5 lineage (19). SphASV1 was more abundant than PseASV1 in both the epiphytic and endophytic fractions of the leaf, and both PseASV1 and SphASV1 were well represented in the endophytic compartment (Fig. 1*C*). SphASV1 was in fact the most abundant single ASV in the dataset. The combination of numerical dominance, endophytic enrichment, and high consistency of colonization drove our interests to characterize genetic and ecological features of this ASV and local *Sphingomonas* more generally.

### *Sphingomonas* Has a Wider Host Range than *Pseudomonas* in the Phyllosphere.

An abandoned train station in Eyach, Germany supports a population of hundreds to thousands of *A. thaliana* plants that are predominantly winter annuals, and has been a source of material for several recent studies of *A. thaliana*-associated microbiomes (6, 8, 10, 19). We revisited this site and collected *A. thaliana* and the most common neighboring plants in spring, when most *A. thaliana* plants were green, mature, and flowering. We also collected common non-*A. thaliana* plants and bulk soil samples in late summer after all local *A. thaliana* vegetation had dried up and died, and the next cohort had not yet germinated. We reasoned that by late summer, microbes that had resided on spring *A. thaliana* must have long since migrated to survive on other plants or habitats (13, 37, 38) or died along with their host, and thus at this later time point, isolates collected from other plant species or soil could not be recent migrants from *A. thaliana*.

We chose each non-*A. thaliana* plant species solely based on its abundance at the site and not by any prior preference for certain plant species, and we therefore chose some plants that we were initially unable to identify, despite being able to confidently classify and recognize different individuals by their morphology. For each plant species, we pooled at least one entire leaf from at least six independent individuals per sample, collecting seven independent samples per species. Although this pooling strategy that spanned many plants per sample precluded analysis of variation between individual plants, it maximized our ability to broadly survey the site (*SI Appendix*, Fig. S1). After bringing leaf samples back to the lab, we surface-sanitized them in 75% ethanol for 45 to 60 s (39) and macerated them in phosphate-buffered saline (PBS) buffer with a mortar and pestle. Soil samples were directly mixed with PBS. We then mixed the fresh lysates with glycerol to make −80 °C freezer stocks (*Methods*).

We extracted metagenomic DNA directly from aliquots of the frozen lysates and prepared 16S rDNA amplicon libraries spanning the V3 and V4 hypervariable regions (*Methods*) using peptide nucleic acids (PNAs) to reduce organelle amplification (40). The residual chloroplast sequences in each sample were consistent within each plant pool, and allowed us to narrow down the identity of the unknown plants (*SI Appendix*, Fig. S2). Unfortunately, we could not obtain sufficient bacterial reads from dandelion and thistle due to a natural mutation in their chloroplast sequences that made our PNAs ineffective (41), leading to an overabundance of chloroplast sequences. To contrast bacterial communities across the sampled plant hosts, we binned the ASVs into bacterial families, and clustered samples by their pairwise Bray–Curtis dissimilarity (Fig. 1*D*). This revealed substantial similarities in bacterial family membership between groups of samples, with clear plant clustering both by sampling season and also by some plant taxa, consistent with previous publications linking plant genotype and seasonal effects to bacterial community composition (14, 42). The family Pseudomonadaceae, of which 92.9% was the genus *Pseudomonas*, reached high relative abundances not only in the *A. thaliana* phyllosphere, but also in some other hosts—particularly the other Brassicaceae *Draba verna* and *Cardamine hirsuta* (Fig. 1*E*). Sphingomonadaceae, of which 80.7% corresponded to the genus *Sphingomonas*, was ubiquitous and abundant across all plant hosts (Fig. 1*E* and *SI Appendix*, Fig. S3). Across all plants, PseASV1 and SphASV1 accounted for a substantial fraction of the reads from *Pseudomonas* and *Sphingomonas* (44.7% and 39.4%, respectively) (Fig. 1 *F* and *G*).

### Cultured Bacterial Populations Resemble Those on Wild Leaves.

*Pseudomonas* isolates from local *A. thaliana* populations in Germany, the majority of which has the PseASV1 16S rDNA sequence, have been previously characterized (19). To investigate *Sphingomonas* genome diversity across host species, and likewise to characterize the genomic features associated with highly abundant *Sphingomonas* groups, we cultured *Sphingomonas* from frozen plant lysates using both remaining *A. thaliana* lysates from ref. (19) as well as from *A. thaliana* plus diverse plant hosts in the present study. We enriched for *Sphingomonas* using Luria Broth (LB) media supplemented with cycloheximide and streptomycin, an antibiotic to which most *Sphingomonas* are resistant due to a natural mutation in the *rpsL* gene (43), and isolated 410 *Sphingomonas* colonies. Using LB supplemented with cycloheximide and nitrofurantoin as previously described (19), we also isolated an additional 103 Pseudomonads. We generated draft genome assemblies, annotated open reading frames, and extracted 16S rDNA sequences (*Methods*). We first analyzed the V3 and V4 regions of the 16S rDNA, which allowed us to match the genomes to our existing culture-independent ASVs. Critically, for both *Pseudomonas* and *Sphingomonas*, we recovered isolates in relative abundances consistent with those from culture-independent surveys (Fig. 1 *F* and *G*), suggesting that culturing did not bias recovery rates and captured broad patterns of diversity on leaves.

### *Sphingomonas* 16S rDNA Sequence Similarity Belies High Genomic Diversity.

**Fig. 2.**
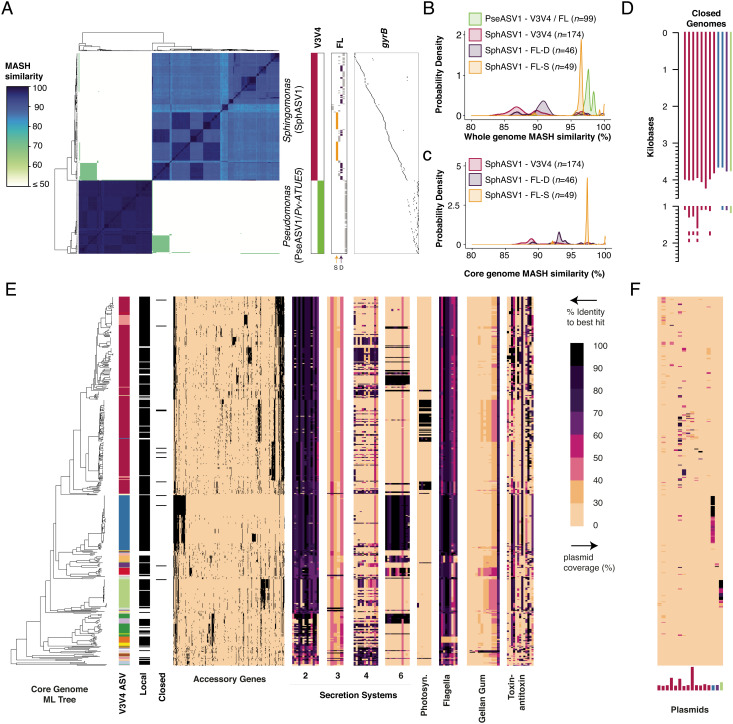
*Sphingomonas* 16S rDNA sequences belie high genomic diversity. (*A*) Heatmap of Mash similarity between 99 *Pseudomonas* PseASV1/Pv-ATUE5 genomes and 174 *Sphingomonas* SphASV1 genomes. The heatmap aligns to the vertical bars to the right, which indicate which genomes contain the V3V4 16S rDNA ASV used to define PseASV1 and SphASV1 ("V3V4," magenta and green), which genomes have different full-length 16S rDNA OTU ("FL"), and which genomes differ in their gyrB fragments ("gyrB"). A diverse group ("D") and a similar group ("S") of genomes sharing a full-length OTU sequence are indicated by arrows underneath the "FL" panel. (*B*) Probability density plot (integral of each curve is 1) showing the distribution of Mash similarities from the heatmap in *A*, subset by bacterial genus and 16S rDNA grouping. V3V4, variable regions 3 and 4; FL, a full-length OTU; FL-D and FL-S, the groups of highly diverse and highly similar genomes, respectively, sharing a full-length OTU sequence as indicated in *A*. (*C*) similar to *B* but showing Mash similarities calculated only on an alignment of 274 core genes. (*D*) Base map of the 12 closed genomes, colored by their V3V4 16S group, showing plasmids to scale. (*E*) Core genome maximum likelihood (ML) tree of 410 *Sphingomonas* isolates from this study and 70 from RefSeq based on 274 core genes, with genomic features indicated by aligned heatmaps. "V3V4," color map of the different V3V4 ASV sequences associated with each genome (white if unknown); "FL OTU," color map of the 99.5% identity OTUs associated with SphASV1 and closely related ASVs; "Local," 410 genomes sequenced and isolated in this study are indicated by black; "Accessory Genes," presence/absence matrix of all genes not used to compute the ML tree; "Secretion Systems," BLAST results of each genome against genes of the type 2, 3, 4, and 6 secretion systems, with percent identities as indicated by the color legend. "Photosyn," genes involved in anoxygenic photosynthesis; "Flagella," genes involved in building an operating flagellum; "Toxin-antitoxin," genes associated with toxin–antitoxin pairs. (*F*) "Plasmids," percent coverage of plasmid sequences from the closed genomes, with the relative size and ASV background of each plasmid indicated by the lengths and colors of the bars.

Previously we observed low genomic diversity among PseASV1/Pv-ATUE5 strains isolated from *A. thaliana* (19). To similarly evaluate genomic diversity for an analogous set of *Sphingomonas*, we selected all the isolates in our collection that both came from *A. thaliana* and had the SphASV1 16S rDNA sequence (representing 174 with SphASV1 out of 340 total *A. thaliana* isolates). We compared the genomes with each other using the Mash algorithm (44), which decomposes genomes into k-mers and calculates a distance based on the fraction of shared k-mers. As a comparator, we also included a representative set of  99 diverse PseASV1-associated genomes isolated from *A. thaliana*, comprising 82 strains from ref. (19) that were at least 0.1% different in Mash distance from all others across the core genome, and an additional group of 17 PseASV1-associated strains from this study. We converted the Mash distances to a similarity score between 0 (least similar) and 100 (identical), which closely corresponds to average nucleotide identity (ANI) (*Methods*) (45, 46). When PseASV1-associated genomes were compared with each other, all pairs had Mash similarities > 96. However, pairwise comparisons between SphASV1-associated *Sphingomonas* genomes had Mash similarities as low as 81 and averaging 89, indicating that the V3V4 region of the 16S rDNA sequence was a relatively poor predictor of genome similarity (Fig. 2 *A* and *B*). Generally, the longer full 16S rDNA sequence (as opposed to one or two variable regions) provides increased discriminatory power between strains (47), and we therefore clustered the full-length 16S rDNA sequences extracted from the genomes into operational taxonomic units (OTUs) at 99.5% identity. This yielded no additional subgroups for PseASV1-associated strains, but partitioned SphASV1-associated strains into six subgroups, five of which included more than one strain and could be compared (Fig. 2*A*). Genomes within these five subgroups were more similar, with average intragroup Mash similarities of 90, 91, 91, 96, and 97. All of these values were lower than for *Pseudomonas* (Fig. 2*B*). We further explored the ability of the gyrase B (gyrB) gene, a commonly used high-resolution phylogenetic marker, to distinguish strains, and extracted gyrB sequences from the assembled genomes (Fig. 2*A*). gyrB outperformed 16S rDNA as a strain-specific marker, and each *Sphingomonas* shared its gyrB sequence with on average 1.6 other SphASV1-associated strains, while each *Pseudomonas* shared its gyrB sequence with on average 4.3 other PseASV1-associated strains.

We initially suspected that the relatively high overall genome diversity of SphASV1-associated isolates compared with PseASV1-associated isolates might be due to high variation in the accessory genomes—specifically the differential presence of plasmids. After closing 12 of our genomes with long-read sequencing, we detected in them a total of 16 plasmids with up to three per genome and comprising up to 14% of the total genome size (Fig. 2*D*). To see if plasmids and mobile elements might be responsible for low *Sphingomonas* genome similarity, we considered only conserved sequences in genomes by first creating a “soft” core genome using panX (48) to identify open reading frames present in at least 70% of genomes, resulting in a set of 274 shared genes. Mash comparisons of core genomes of SphASV1-associated isolates (Fig. 2*C*) were still more diverse than even whole genomes of PseASV1-associated isolates. Thus, despite the pervasiveness of the SphASV1 sequence in our dataset, one cannot reliably extrapolate what this ASV sequence means at the level of genomic content.

### *Sphingomonas* Genomes Reveal Adaptations for Competitive Life in the Phyllosphere.

To explore relatedness of SphASV1-associated strains, and how they compare with other *Sphingomonas*, we calculated maximum-likelihood (ML) “soft” core genome phylogenies (48) from all 410 local *Sphingomonas* isolates (340 from *A. thaliana* and 70 from other local plant hosts), along with 70 sequence-related isolates from NCBI RefSeq (49). Gene presence or absence in the accessory genome (Fig. 2*E*) tracked well with differences in the core genome, as tested by correlating pairwise Jaccard distances calculated on the presence/absence matrix of accessory genes to Mash distances calculated on core genes (R^2^ = 0.86, *SI Appendix*, Fig. S4). We compiled a short list of *Sphingomonas* genes that had a high likelihood to improve survival among competing bacteria, or to facilitate interaction with a plant host, and made a custom database of *Sphingomonas* protein sequences from RefSeq (Dataset S1). We searched for these features in our genomes by aligning the assemblies using BLASTX (50). While nearly all isolates have diagnostic genes for the type 2 secretion system, and genes for the type 4 and type 6 secretion systems are common among some clades including those of SphASVI, only a handful of less abundant strains seems to potentially have a type 3 secretion system (Fig. 2*E*). Flagellar motility is common. A group of SphASV1 genomes has a full suite of genes for anoxygenic photosynthesis, a fascinating feature that can supplement heterotrophic energy production and likely improves survival in well-illuminated and nutrient-poor conditions such as the phyllosphere (51, [Bibr r52], [Bibr r53]–54). All genomes are rife with toxin–antitoxin systems, likely to stabilize plasmids or superintegrons (55). Indeed, we found widespread evidence of plasmids in our draft genomes, with 250 (60%) of our isolates showing signatures of one or more of the 16 plasmids identified in the closed genomes (Fig. 2*F*).

To increase confidence in the preceding gene searches in our draft genomes, we repeated the analysis comparing the 12 closed genomes to their draft genome counterparts. Both draft and complete versions of each genome showed essentially the same presence/absence patterns (*SI Appendix*, Fig. S5), demonstrating that draft genomes were sufficient for analysis of gene content at this level of detail.

### Some *Sphingomonas* Attenuate *Pseudomonas* Virulence in *A. thaliana*.

Besides reaching similarly high abundances in the same leaves (Fig. 1 *A* and *B*), both *Sphingomonas* and *Pseudomonas* grow on many similar substrates in vitro (35), making it likely they interact in the phyllosphere. Previous work revealed that certain strains of *Sphingomonas*, in particular *Sphingomonas melonis* Fr1, can ameliorate symptoms in *A. thaliana* leaves caused by pathogenic *Pseudomonas* and *Xanthomonas* in a gnotobiotic system (24, 35, 56). More recently, a strain of *S. melonis* was demonstrated to protect rice against the bacterial seedling blight pathogen *Burkholderia plantarii* (22). We first sought to screen some of our local isolates for potential plant-protective activity against virulent Pv-ATUE5 strains in a gnotobiotic system as we used previously (19).

**Fig. 3.**
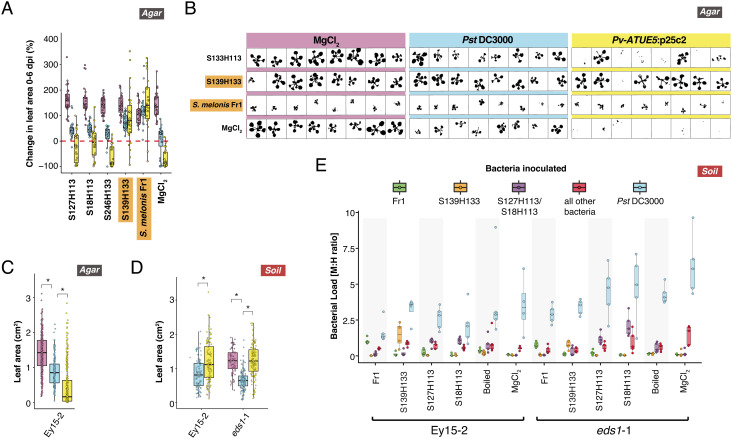
Some *Sphingomonas* offer protection against *Pseudomonas*. (*A*–*C*), Local *Sphingomonas* isolates, *S. melonis* Fr1 (Fr1), or MgCl2 buffer were used to pretreat *A. thaliana* Ey15-2 seeds germinating in 24-well agar plates. On day 10, seedlings were challenged with a MgCl2 buffer control, Pst DC3000, or Pv-ATUE5:p25c2 and monitored for 6 d after infection (n ≥ 6 per condition). Orange-highlighted *Sphingomonas* insignificant difference between infected and buffer-treated plants (Mann–Whitney *U* test with FDR adjustment, *P* > 0.05). Boxes enclose the interquartile range (IQR) with whiskers extending to up to 1.5 times the IQR. (*A*) Percent change in rosette size between 0 and 6 dpi. (*B*) Representative silhouettes at 6 dpi of seedlings grown in 24-well plates. (*C*) Rosette size by infection treatment, regardless of *Sphingomonas* pretreatment. *P < 0.001 (Mann–Whitney *U* test with FDR adjustment). (*D* and *E*) Three local *Sphingomonas* isolates, *S. melonis* Fr1, boiled *Sphingomonas*, or buffer were sprayed on 2-wk-old seedlings of Ey15-2 or eds1-1, and 4 d later, live *Pseudomonas* or MgCl2 buffer was inoculated. d, Absolute sizes of rosettes for different *Pseudomonas* treatments, regardless of *Sphingomonas* treatment. *P < 0.001 (Mann–Whitney *U* test with FDR adjustment). (*E*) Bacterial load ratio in plants infected with Pst DC3000 as determined by hamPCR. Note that the color legend represents bacteria quantified, not the bacteria used for treatment. Boxes enclose the IQR with whiskers extending to up to 1.5 times the IQR.

We germinated an *A. thaliana* accession endemic to our field site, Ey15-2, on half-strength MS solid media in the presence of each of 19 diverse strains of local *Sphingomonas*, as well as *S. melonis* Fr1. Following 10 d of cocultivation, we challenged the plants with *Pseudomonas*, either the virulent local *P. viridiflava* strain Pv-ATUE5:p25c2 (19) or the model pathogen *P. syringae pv. tomato* (*Pst*) DC3000, and monitored plant health over the next 6 d by measuring green pixels (*Methods* and Dataset S2). Pst DC3000 slowed plant growth compared with buffer control, while Pv-ATUE5:p25c2 killed plant tissues (Fig. 3 *A*–*C* and *SI Appendix*, Fig. S6). Surprisingly, seedlings germinated in the presence of *S. melonis* Fr1 were consistently stunted compared with all other plants (Fig. 3*B* and *SI Appendix*, Fig. S6 and Discussion S1). However, despite this negative effect on growth, *S. melonis* Fr1 protected plants from the worst effects of Pv-ATUE5:p25c2, with infected plants not dying but instead growing more slowly, not significantly different from plants treated with the less lethal Pst DC3000 across two replicates (FDR-adjusted Mann–Whitney *U* test, *P* > 0.05). Our local *Sphingomonas* strain SphATUE:S139H133 also protected plants, reducing Pv-ATUE5:p25c2 virulence such that it was no worse than Pst DC3000 across two independent experiments (Fig. 3 *A* and *B* and *SI Appendix*, Fig. S6) (FDR-adjusted Mann–Whitney *U* test, *P* > 0.05). Unlike *S. melonis* Fr1, SphATUE:S139H133 did not stunt growth.

To test whether protective effects might extend to more natural conditions, we grew plants on potting soil, and set up a similar experiment in which 2-wk-old Ey15-2 seedlings were sprayed first with one of four *Sphingomonas* strains or a boiled *Sphingomonas* control, and 4 d later were challenged with *Pseudomonas* sprayed at high concentrations (O.D.600 = 1.0) (57). We also included enhanced disease susceptibility 1 (eds1-1, Ws-0 background) as an infection control because this mutant, defective for numerous defense responses mediated by salicylic acid (SA), is hypersusceptible to Pst DC3000 (58). At 5 d post infection (dpi), we observed classic Pst DC3000 symptoms on most plants including chlorotic leaves, stunted growth, and increased anthocyanin at the apical meristem (57), especially on eds1-1 plants. However Pv-ATUE5:p25c2, which was consistently deadly on agar plates, did not produce any obvious symptoms on soil-grown *A. thaliana* and did not greatly affect rosette size (Fig. 3*D* and *SI Appendix*, Discussions S2 and Fig. S8).

In the protection experiment on Ey15-2 and eds1-12 plants, we did not observe a consistent protective effect of any local *Sphingomonas* strain against Pst DC3000 symptoms on soil. However, for both Ey15-2 and eds1-1 plants, pre-treatment with *S. melonis* Fr1 did result in larger plants than pretreatment with boiled *Sphingomonas* or with buffer (FDR-adjusted Mann–Whitney *U* test, *P* < 0.05, *SI Appendix*, Fig. S7). In contrast to the agar system, *S. melonis* Fr1-treated Ey15-2 plants grown on soil were not stunted (*SI Appendix*, Fig. S7). To confirm that *Sphingomonas* was still present at the end of the experiment, and if pathogen titers had been affected by *Sphingomonas*, we quantified bacterial communities in the leaves of eight plants per condition at 5 dpi using hamPCR (59). We observed the inoculated *Sphingomonas* 16S rDNA sequences enriched in each end point sample, as expected (Fig. 3*E*). Samples pre-treated with *S. melonis* Fr1 supported less Pst DC3000 proliferation than those pretreated with other *Sphingomonas* or buffer in both the Ey15-2 and eds1-1 backgrounds, although Fr1 pretreatment only significantly differed from buffer in eds1-1 (Mann–Whitney *U* test, *P* < 0.05). However, we found no evidence that other isolates, including the local Sph139H133, which was protective on agar, reduced pathogen titers in plants grown on soil in these strong infection conditions.

### *Sphingomonas* and *Pseudomonas* Strains Thrive on Multiple Plant Species.

**Fig. 4.**
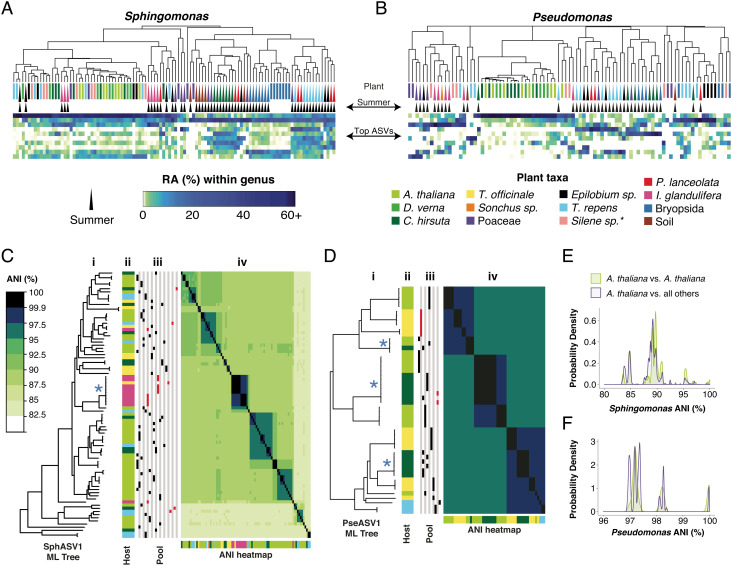
Isolates from other plant species match those isolated from *A. thaliana*. (*A* and *B*) Host species (*Top*) from 2018 clustered by Bray–Curtis dissimilarities between relative abundances (RA) for the 10 most abundant 16S rDNA ASVs for each genus. The legend below the panels is shared for both panels. A * represents uncertainty in the plant taxon name. (*A*) *Sphingomonas* ASVs, with SphASV1 as the top row in the heatmap. (*B*) Same as *A*, but for *Pseudomonas* ASVs. (*C*) Heatmap revealing genome ANI of all pairwise comparisons between SphASV1 isolates. i: Isolates arranged by a maximum likelihood (ML) core genome tree. The asterisk approximately in the middle of the tree represents the position of a group of isolates sharing >99.9% ANI that derives from different sample pools, as indicated in columns (iii) to the right. ii: Plant host of origin is indicated by color, corresponding to the plant legend in *A* and *B*. iii: Isolates from the same sample pool are indicated by red (summer) or black (spring) marks in the same vertical column. iv: heatmap showing pairwise ANI values. (*D*), Same as *C*, but for PseASV1. (*E*) Distribution of pairwise intrahost isolate ANI compared to interhost isolate ANI for SphASV1. (*F*) Same as *E*, but for PseASV1 isolates.

*Sphingomonas* and *Pseudomonas* are generalists, with no known exclusive hosts of any given strain (13). As a first step toward determining the breadth of hosts in our study area, we compared *Sphingomonas* and *Pseudomonas* ASVs across local plant species. While SphASV1 was more abundant overall in the spring collection, it was consistently detectable on most plant species in both seasons. Plant taxa colonized by SphASV1 were also frequently colonized to appreciable levels by multiple other *Sphingomonas* ASVs (Fig. 4*A*). In contrast, PseASV1 was relatively more abundant on *A. thaliana*, *C. hirsuta* and *D. verna* – all from the family Brassicaceae – and less abundant, though still easily detectable, on other taxa (Fig. 4*B*). PseASV1 was especially enriched in the spring, which matches our previous finding of more Pv-ATUE5 isolates from spring vs. winter collected *A. thaliana* (19).

To test for strain sharing across hosts, we compared PseASV1 and SphASV1 genomes of isolates from *A. thaliana*, *T. officinale*, *T. repens*, *C. hirsuta*, and *I. glandulifera* (Fig. 4 *C* and *D*). These species were chosen because we had been able to easily isolate both *Pseudomonas* and *Sphingomonas* from the same plant pool lysates prepared for each of these species. We compared the genomes’ ANI(45, 46). While 29 out of the 86 SphASV1 isolates from the 2018 harvest shared at least 99.9% ANI with at least one other isolate, with only one exception these highest similarity isolates came from the same pool of plants, implying that there was little evidence for the same clonal *Sphingomonas* lineage independently colonizing different plant individuals. To exclude potential clones from the same plant, we recalculated all pairwise genome similarities between SphASV1 isolates from different lysates. We compared similarity within *A. thaliana* isolates (intrahost isolate similarity) and between *A. thaliana* isolates and those from any other plant species (interhost isolate similarity). A higher intrahost similarity would be evidence of host-specificity, possibly due to unique selective forces within *A. thaliana*, or easier migration between individuals. We found that the distribution of intrahost ANI values closely matched that of interhost ANI values (Fig. 4*E*), with the intrahost ANI being only marginally higher (0.9%, Mann–Whitney *U* test, *P* < 0.001).

PseASV1 genomes were more similar to each other than SphASV1 genomes (Fig. 2*A*). Every isolate shared at least 97.5% ANI with at least one isolate from a different plant species, and 47 of the 50 isolates shared at least 99.9% ANI with at least one other isolate (Fig. 4*D*), although as with SphASV1, these highest similarity isolates tended to come from the same plant pool. However, three groups of highest similarity isolates were shared across different plant pools, different species, and even different seasons (Fig. 4*D*). This is consistent with previous observations that Pv-ATUE5 strains are common and persistent on diverse *A. thaliana* populations (19), and demonstrates that other local plant species host these strains as well. As with SphASV1 isolates, the intrahost ANI values followed a similar distribution to interhost ANI values, with intrahost isolate ANI values again being marginally higher (0.4%, Mann–Whitney *U* test, *P* < 0.001, Fig. 4*F*).

### Bulk Culture Metagenomics Reveals Strain Sharing Across Plant Species.

**Fig. 5.**
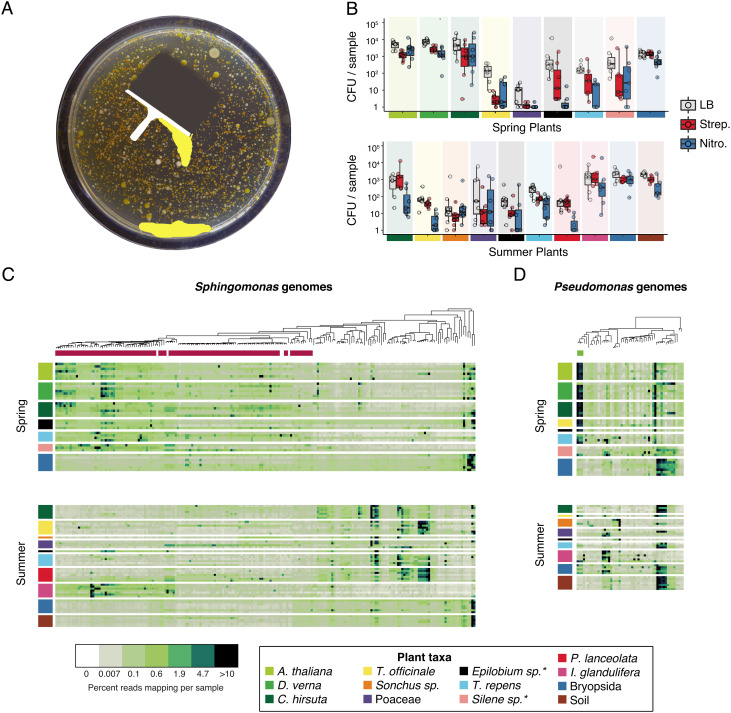
Metagenomics of cultured bacteria reveals strain sharing across plant species. (*A*) Cartoon demonstrating the process of generating and harvesting bulk cultures for plant-free metagenomics. (*B*) Colony forming units for each plant host species (see color legend, *Bottom Left*) on medium containing no antibiotic (LB), *Sphingomonas*-selective streptomycin (Strep) medium, or *Pseudomonas*-selective nitrofurantoin (Nitro) medium. Each sample represents about 4.5 mg fresh weight of original leaf material. Boxes enclose the interquartile range (IQR) with whiskers extending to up to 1.5 times the IQR. (*C*) Heatmap showing fourth-root transformed reads from each *Sphingomonas* bulk culture metagenome (rows, colors from plant host taxa shown in the legend below) that map to a *Sphingomonas* local reference genome (columns), for the spring collection (*Top*) and the summer collection (*Bottom*). A * represents uncertainty in the plant taxon name. The genetic relatedness of the local bacterial reference genomes is shown by a maximum likelihood (ML) tree above the heatmap. Those reference genomes belonging to SphASV1 are indicated under the ML tree in magenta. Darker colors in the heatmap correspond to genomes attracting a greater fraction of reads in the mapping process. (*D*) Same as c, but showing *Pseudomonas* bulk culture mapping to *Pseudomonas* reference genomes. Reference genomes belonging to PseASV1 are indicated under the ML tree in green.

From our genome-sequenced isolates, we had found that closely related *Sphingomonas* and *Pseudomonas* strains could colonize diverse hosts. To broaden our survey and extend these observations, we adopted a time and cost-efficient approach to enrich each genus in bulk from plant lysates and sequence the enriched pool as a metagenome. We plated glycerol stock from each lysate on either selective *Pseudomonas* or *Sphingomonas* medium and grew colonies en masse (Fig. 5*A* and *SI Appendix*, Fig. S9). As a control for lysate viability and a rough reference of bacterial load, we also cultured bacteria from lysates on nonselective rich LB medium. We counted the colonies on each plate after 2 d at room temperature for *Pseudomonas* or mixed bacteria on LB, or after 7 d for slower growing *Sphingomonas* (Fig. 5*B*) (*Methods*).

To harvest the bacteria, we scraped all colonies from each plate and prepared whole metagenome shotgun libraries from the pools (Fig. 5*A*). After sequencing the metagenomes, we mapped the reads to a comprehensive reference database including all of our local genomes plus selected publicly available genomes (49, 60) (*Methods* and *SI Appendix*, Figs. S10–S12). Because some colonies did not belong to our targeted genera, we also included in our reference database “decoy” genomes of common plant-associated bacteria to capture reads from these “contaminant” strains (*SI Appendix*, Fig. S11). A total of 165 metagenomes (91 *Sphingomonas* + 74 *Pseudomonas*) passed our quality thresholds. For both bacterial genera, there was a clear shift in dominant strains across seasons, including for *C. hirsuta* and *T. repens*, which were alive and were sampled at both timepoints (Fig. 5 *C* and *D*). This seasonal shift was also apparent in the *Sphingomonas* and *Pseudomonas* amplicon data (Fig. 4 *A* and *B*). Finally, many metagenome reads from diverse plant samples mapped to the same strains, in agreement with them being widely shared across plants. In particular, this could be observed for strains associated with the most abundant 16S rDNA sequences, SphASV1 and PseASV1. The relative representation of both SphASV1 and PseASV1 isolates in the summer soil samples was low compared with that in plants, suggesting that as the new cohort of *A. thaliana* germinates in the following fall, it may be more likely that *A. thaliana* seedlings acquire these strains from nearby plants rather than from the soil.

### *Pseudomonas* Are Much Stronger Leaf Saprophytes than *Sphingomonas*.

**Fig. 6.**
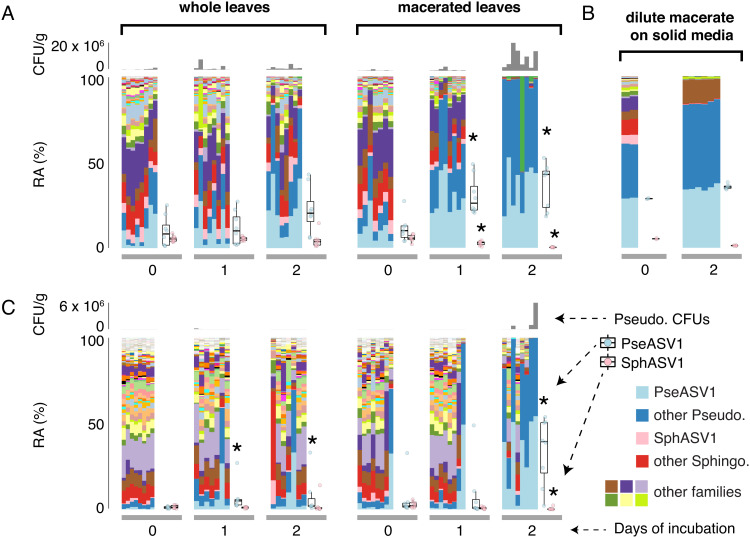
Contrasting *Pseudomonas* and *Sphingomonas* growth in whole vs macerated leaves. (*A*), Bacteria growing in wild *A. thaliana* whole leaves (*Left*) or macerated leaves (*Right*) at 0, 1, and 2 d post harvesting. The layout for all panels is as diagrammed on the *Right* of panel (*C*), with *Pseudomonas* CFU/g shown as a gray column chart (*Top*), the relative abundance (RA) of different bacterial families shown as a stacked barplot colored according to the legend, and with the relative abundances of PseASV1 and SphASV1 for each timepoint summarized as box plots. An asterisk (*) represents a significant difference from 0 d of incubation (*P* < 0.05 according to a Mann–Whitney *U* test). (*B*) Similar to *A*, but with bacteria from macerated *A. thaliana* leaves at the time of harvest and after 2 d of incubation on LB plates. *Pseudomonas* CFUs were not counted (*C*), Same as *A*, but with bacteria in wild *B. napus* leaves instead of *A. thaliana*.

We consistently noticed a slower growth rate for *Sphingomonas* than for *Pseudomonas*, but SphASV1 seemed to be as successful as PseASV1 in establishing substantial population sizes in leaves of wild plants. We hypothesized that the LB medium might advantage *Pseudomonas*, and growth of the bacteria in the more complex nutrient and chemical milieu supplied by plants might result in more equal performance between the genera. To test this, we collected wild leaves of both *A. thaliana* and Brassica napus from a site in Kusterdingen, Germany in May 2021. For both *A. thaliana* and *B. napus* leaves, PseASV1 and SphASV1 were present and highly abundant in all starting leaf material. We macerated a subset of the leaves with a mortar and pestle, and then compared the growth of bacteria over the next 2 d within macerated leaves and within detached but unmacerated control leaves (Fig. 6), with all samples kept in a plant growth chamber. At each timepoint, we quantified bacterial relative abundances with 16S rDNA amplicon sequencing to detect all genera, and we cultivated and counted *Pseudomonas* CFUs to estimate changes in absolute bacterial abundances.

PseASV1 proliferated to represent a strong majority of bacteria in most *A. thaliana* and *B. napus* leaf macerates (Fig. 6 *A* and *C*). While PseASV1 also increased its relative abundance in detached whole leaves of both plant species, the magnitude was much less pronounced than in macerates. We also plated liquid collected from *A. thaliana* leaf macerate onto solid LB agar and incubated it in the same conditions and examined the bacterial population after 2 d (Fig. 6*B*). As in macerated plant tissue, PseASV1 strains on LB markedly increased their abundances in the community after 2 d, while the relative abundance of SphASV1 strains and indeed all Sphingomonadaceae decreased to essentially zero (Fig. 6*B*). These results strongly suggest that PseASV1 thrives in dead or dying leaves and furthermore on rich nonliving substrates, with the intriguing corollary being that *Sphingomonas* depends on healthy leaves to maintain its competitive edge.

## Discussion

Most microbes that live in or on multicellular organisms do not have an obligate relationship with any specific multicellular species, but rather are better adapted to some, which are called hosts, than to others, which are called nonhosts (61, 62). We sought to use genomic and metagenomic techniques to characterize, at the strain level, the extent of host specialization for the two bacterial genera that are locally the most abundant in the phyllosphere of the host plant *A. thaliana*. We found a higher genomic diversity of *Sphingomonas*, which generally reaches very similar population sizes in the leaves of our plant populations, than for *Pseudomonas*, which has very different population sizes in different individuals. The high genomic diversity of *Sphingomonas* held true even among those sharing the same 16S rDNA sequence, highlighting the importance of strain-resolved techniques also in wild ecosystems. To this end we employed direct culturing and sequencing of individual isolates, as well as bulk-culture metagenomics (Fig. 5). While culturing is less quantitative and potentially less inclusive than direct sequencing, genetically diverse *Sphingomonas* and *Pseudomonas* can be cultured with low bias (Fig. 1 *F* and *G*), making this a powerful technique to further reveal colonization patterns for strains in these genera.

Our previous work characterizing PseASV1/Pv-ATUE5 suggested that much of its standing genetic variation predates *A. thaliana* colonizing SW Germany (19), and we had puzzled how ancient variants are apparently able to continue to coinfect the entire population in this pathosystem because in agricultural systems epidemics are typically monomorphic with a rapid turnover of pathogenic isolates within a few years (63, 64). The present study begins to answer this question. First, PseASV1/Pv-ATUE5 strains efficiently colonize other local plant species besides *A. thaliana*, especially other Brassicaceae. The same *Pseudomonas* strains observed on *A. thaliana* also persist through the summer, living on other hosts when there are no alive *A. thaliana* plants, implying that their performance on these additional hosts may be equally, if not more important to their long-term success than their seasonal exploitation of *A. thaliana* as a host. Currently, we do not know how important different hosts are for different subclades of PseASV1/Pv-ATUE5, but with a variety of host species as well as intraspecific genetic heterogeneity in each host, it may be difficult for any one strain to prevail and dominate the entire *Pseudomonas* population – matching a scenario of diffuse interactions, as proposed before as an explanation for nonmatching distributions of a specific *Pseudomonas* syringae effector and the *A. thaliana* resistance locus that detects it (65). In contrast, in a crop monoculture, genetically identical hosts provide more consistent and uniform challenges for a pathogen, making it more likely for a single strain to become dominant. A second explanation for the lack of a single emergent strain is that the pathogenic nature of PseASV1/Pv-ATUE5 is strongly context dependent, and apparently more so than for other *Pseudomonas* pathogens such as Pst DC3000. In an agar plate system, disease caused by PseASV1/Pv-ATUE5 was much more severe than that of Pst DC3000, but in potting soil, PseASV1/Pv-ATUE5 failed to cause significant disease symptoms and grew much more slowly than Pst DC3000. Consistent with fitness effects of PseASV1/Pv-ATUE5 being greatly modulated by the environment, we saw few disease symptoms on field-collected plants at the time of collection, regardless of their *Pseudomonas* load.

Compared with PseASV1/Pv-ATUE5, *Sphingomonas* strains that shared the most abundant V3V4 *Sphingomonas* ASV, SphASV1, were more than three times as diverse by ANI in their core genomes. SphASV1 isolates differed in type 4 and type 6 secretion system presence, anoxygenic photosynthetic ability, plasmid presence, and in other unidentified features that are likely to affect host colonization potential or intermicrobial competition. The plasmid count, up to three per strain in our closed genomes, suggests that the *Sphingomonas* genetic tool kit may be highly modular. We observed SphASV1 in diverse 16S rDNA datasets worldwide, including high abundances on Boechera stricta in North America (14), and while the extent of genome variation among SphASV1 strains in SW Germany makes it difficult to predict genomic features from 16S rDNA, it will be highly interesting to determine the global diversity in this group of *Sphingomonas* strains.

This study also demonstrated that apart from SphASV1, the genus *Sphingomonas* is not only abundant, but consistently so across plants (Fig. 1). It colonizes diverse plants at our field site to high levels (*SI Appendix*, Fig. S3), a result consistent with reports of high abundances on leaves of diverse plants such as maize (15), poplar (11, 12), and other species (11). The different colonization patterns of the genera *Pseudomonas* and *Sphingomonas* provide clues about their lifestyle strategies. The fact that *Pseudomonas* populations vary greatly in size between plants, occasionally reaching high abundances as homogenous blooms of PseASV1/Pv-ATUE5 (19), suggests that different strains may compete for resources and share the same niche, making stable coexistence within a plant less likely. In contrast, *Sphingomonas* loads varied little across plants, regardless of the load of other bacteria (Fig. 1*A*), and the diversity of *Sphingomonas* ASVs in each plant was also higher. The more balanced coexistence of different *Sphingomonas* strains may mean that each occupies a different microniche, or that *Sphingomonas* have means to limit their population growth, such as for example a quorum sensing system shared by the genus. To what extent these bacteria inhabit spatially distinct parts of the leaf or grow as biofilms is unknown; future direct visualization techniques will help resolve this.

Some *Sphingomonas* can project against pathogenic *Pseudomonas* and other bacterial and fungal pathogens, and they also affect overall microbial community structure (6, 33). This may be in part because their metabolic needs substantially reduce the availability of substrates for other phyllosphere microbes (35). However, in at least one known case, a *Sphingomonas* strain secretes an extracellular small molecule that attenuates the virulence of a bacterial pathogen (22). Further, the plant protective *S. melonis* Fr1 induces transcriptional changes in *A. thaliana* and protection depends on the presence of a plant immune receptor (56). These examples illustrate that protective mechanisms go beyond substrate competition. Although we estimate that in our culture collection only a minority of *Sphingomonas* strains have protective ability against PseASV1/Pv-ATUE5 or Pst DC3000, the high bacterial titers we inoculated with and the gnotobiotic environment in which we observed the strongest effects may limit transferability of our results to the field, especially as the most protective local *Sphingomonas* isolate had a 16S rDNA sequence that was not among the more abundant on field plants. The high diversity of *Sphingomonas* genomes, the presence of secretion systems often involved in biotic interactions, and the fact that we observed some antagonism suggest there is more to be discovered. The overall implications of *Sphingomonas* populations on leaves will be important to understand, both for their direct impacts on plant recognition and tolerance of nonpathogens and also for their role in structuring wild microbial communities.

The evolutionary pressures on these generalist bacteria are many (66), involving at a minimum multiple host plants with different immune system activities as well as free-living phases. Our final experiment demonstrating PseASV1-associated strains’ growth advantage over SphASV1-associated strains in detached leaves, macerated leaves, and solid media hints at the fact that very different growth strategies drive dominance in the phyllosphere. *Sphingomonas* grows more slowly and appears to thrive in healthy leaves, perhaps because of a higher investment in defense against the plant, and we speculate that investing in plant-beneficial features may be part of a strategy to coopt the plant’s immune system to help prevent opportunists or saprophytes from outcompeting it. In contrast, *Pseudomonas* strains quickly overtake weakened and dead leaves, and while they may survive on healthy leaves, occasionally weakening the plant immune system can promote greater populations and bacterial spread.

## Materials and Methods

Wild plant samples were collected from southern Germany and were used for both direct DNA sequencing and for culturing *Sphingomonas* and *Pseudomonas*. Bacteria cultures were isolated and propagated on solid agar media with appropriate antibiotics. All DNA was extracted using stringent bead beading to ensure lysis of microbial cells. Bacterial genomes were sequenced and assembled using established methods. Pangenome and phylogenetics analysis were accomplished with panX (48). Full details of the methods used in this work are described in **SI Appendix*, Materials and Methods*. Scripts used for custom computational methods are available at: https://github.com/derekLS1/ContrastingPatternsDominance.

## Supplementary Material

Appendix 01 (PDF)

Dataset S01 (XLSX)

Dataset S02 (XLSX)

Dataset S03 (XLSX)

Dataset S04 (XLSX)

## Data Availability

All sequence data in this manuscript are deposited with the European Nucleotide Archive (ENA) under project number PRJEB44136 (67) https://www.ebi.ac.uk/ena/browser/view/PRJEB44136. The ENA accession numbers for individual raw reads and assemblies can be found in Dataset S3.

## References

[1] M. L. Avolio , Demystifying dominant species. New Phytol. **223**, 1106–1126 (2019). 30868589 10.1111/nph.15789

[r2] Y. Zhou , Biogeography of the ecosystems of the healthy human body. Genome Biol. **14**, R1 (2013). 23316946 10.1186/gb-2013-14-1-r1 PMC4054670

[3] B. J. McGill , Species abundance distributions: Moving beyond single prediction theories to integration within an ecological framework. Ecol. Lett. **10**, 995–1015 (2007). 17845298 10.1111/j.1461-0248.2007.01094.x

[4] S. L. Heaver, E. L. Johnson, R. E. Ley, Sphingolipids in host-microbial interactions. Curr. Opin. Microbiol. **43**, 92–99 (2018). 29328957 10.1016/j.mib.2017.12.011

[5] A. G. Wexler, A. L. Goodman, An insider’s perspective: Bacteroides as a window into the microbiome. Nat. Microbiol. **2**, 17026 (2017). 28440278 10.1038/nmicrobiol.2017.26 PMC5679392

[6] M. T. Agler , Microbial hub taxa link host and abiotic factors to plant microbiome variation. PLoS Biol. **14**, e1002352 (2016). 26788878 10.1371/journal.pbio.1002352 PMC4720289

[7] J. A. Vorholt, Microbial life in the phyllosphere. Nat. Rev. Microbiol. **10**, 828–840 (2012). 23154261 10.1038/nrmicro2910

[8] T. L. Karasov , The relationship between microbial biomass and disease in the *Arabidopsis thaliana* phyllosphere. bioRxiv [Preprint] (2019). 10.1101/828814. Accessed 08 April 2020.

[9] N. Bodenhausen, M. W. Horton, J. Bergelson, Bacterial communities associated with the leaves and the roots of *Arabidopsis thaliana*. PLoS One **8**, e56329 (2013). 23457551 10.1371/journal.pone.0056329 PMC3574144

[10] J. Regalado , Combining whole-genome shotgun sequencing and rRNA gene amplicon analyses to improve detection of microbe–microbe interaction networks in plant leaves. ISME J. **14**, 2116–2130 (2020). 32405027 10.1038/s41396-020-0665-8 PMC7368051

[11] H. Kim , High population of *Sphingomonas* species on plant surface. J. Appl. Microbiol. **85**, 731–736 (1998).

[12] J. Massoni , Consistent host and organ occupancy of phyllosphere bacteria in a community of wild herbaceous plant species. ISME J. **14**, 245–258 (2019), 10.1038/s41396-019-0531-8. 31624344 PMC6908658

[13] C. E. Morris, J. R. Lamichhane, I. Nikolić, S. Stanković, B. Moury, The overlapping continuum of host range among strains in the *Pseudomonas* syringae complex. Phytopathol. Res. **1**, 4 (2019).

[14] M. R. Wagner , Host genotype and age shape the leaf and root microbiomes of a wild perennial plant. Nat. Commun. **7**, 12151 (2016). 27402057 10.1038/ncomms12151 PMC4945892

[15] J. G. Wallace, K. A. Kremling, L. L. Kovar, E. S. Buckler, Quantitative genetics of the maize leaf microbiome. Phytobiomes J. **2**, 208–224 (2018).

[16] K. L. Grady, J. W. Sorensen, N. Stopnisek, J. Guittar, A. Shade, Assembly and seasonality of core phyllosphere microbiota on perennial biofuel crops. Nat. Commun. **10**, 4135 (2019). 31515535 10.1038/s41467-019-11974-4 PMC6742659

[17] C.-J. Dong, L.-L. Wang, Q. Li, Q.-M. Shang, Bacterial communities in the rhizosphere, phyllosphere and endosphere of tomato plants. PLoS One **14**, e0223847 (2019). 31703074 10.1371/journal.pone.0223847 PMC6839845

[18] R. S. C. de Souza , Unlocking the bacterial and fungal communities assemblages of sugarcane microbiome. Sci. Rep. **6**, 28774 (2016). 27358031 10.1038/srep28774 PMC4928081

[19] T. L. Karasov , *Arabidopsis thaliana* and *Pseudomonas* pathogens exhibit stable associations over evolutionary timescales. Cell Host Microbe **24**, 168–179.e4 (2018). 30001519 10.1016/j.chom.2018.06.011 PMC6054916

[20] H. C. McCann , Genomic analysis of the Kiwifruit pathogen *Pseudomonas* syringae pv. actinidiae provides insight into the origins of an emergent plant disease. PLoS Pathog. **9**, e1003503 (2013). 23935484 10.1371/journal.ppat.1003503 PMC3723570

[21] T. C. Helmann, A. M. Deutschbauer, S. E. Lindow, Genome-wide identification of *Pseudomonas* syringae genes required for fitness during colonization of the leaf surface and apoplast. Proc. Natl. Acad. Sci. U.S.A. **116**, 18900–18910 (2019). 31484768 10.1073/pnas.1908858116 PMC6754560

[22] H. Matsumoto , Bacterial seed endophyte shapes disease resistance in rice. Nat. Plants **7**, 60–72 (2021). 33398157 10.1038/s41477-020-00826-5

[23] N. C. Snelders , Microbiome manipulation by a soil-borne fungal plant pathogen using effector proteins. Nat. Plants **6**, 1365–1374 (2020). 33139860 10.1038/s41477-020-00799-5

[24] C. Vogel, G. Innerebner, J. Zingg, J. Guder, J. A. Vorholt, Forward genetic in planta screen for identification of plant-protective traits of *Sphingomonas* sp. strain Fr1 against *Pseudomonas* syringae DC3000. Appl. Environ. Microbiol. **78**, 5529–5535 (2012). 22660707 10.1128/AEM.00639-12 PMC3406163

[25] J. L. W. Thudichum, A Treatise on the Chemical Constitution of the Brain (Baillière & Company, 1884).

[26] U. Ali, H. Li, X. Wang, L. Guo, Emerging roles of sphingolipid signaling in plant response to biotic and abiotic stresses. Mol. Plant **11**, 1328–1343 (2018). 30336328 10.1016/j.molp.2018.10.001

[27] E. L. Johnson , Sphingolipids produced by gut bacteria enter host metabolic pathways impacting ceramide levels. Nat. Commun. **11**, 2471 (2020). 32424203 10.1038/s41467-020-16274-w PMC7235224

[28] Y. K. Yeoh , Evolutionary conservation of a core root microbiome across plant phyla along a tropical soil chronosequence. Nat. Commun. **8**, 215 (2017). 28790312 10.1038/s41467-017-00262-8 PMC5548757

[29] H. R. Barajas , Testing the two-step model of plant root microbiome acquisition under multiple plant species and soil sources. Front. Microbiol. **11**, 542742 (2020). 33162946 10.3389/fmicb.2020.542742 PMC7581803

[30] F. O. Aylward , Comparison of 26 Sphingomonad genomes reveals diverse environmental adaptations and biodegradative capabilities. Appl. Environ. Microbiol. **79**, 3724–3733 (2013). 23563954 10.1128/AEM.00518-13 PMC3675938

[31] S. Asaf, M. Numan, A. L. Khan, A. Al-Harrasi, *Sphingomonas*: From diversity and genomics to functional role in environmental remediation and plant growth. Crit. Rev. Biotechnol. **40**, 138–152 (2020). 31906737 10.1080/07388551.2019.1709793

[32] A. L. Khan , Bacterial endophyte *Sphingomonas* sp. LK11 produces gibberellins and IAA and promotes tomato plant growth. J. Microbiol. **52**, 689–695 (2014). 24994010 10.1007/s12275-014-4002-7

[33] C. I. Carlström , Synthetic microbiota reveal priority effects and keystone strains in the Arabidopsis phyllosphere. Nat. Ecol. Evol. **3**, 1445–1454 (2019). 31558832 10.1038/s41559-019-0994-z PMC6774761

[34] N. C. Snelders , Microbiome manipulation by a soil-borne fungal plant pathogen using effector proteins. Nat. Plants **6**, 1365–1374. 10.1038/s41477-020-00799-5 33139860

[35] G. Innerebner, C. Knief, J. A. Vorholt, Protection of *Arabidopsis thaliana* against leaf-pathogenic *Pseudomonas* syringae by *Sphingomonas* strains in a controlled model system. Appl. Environ. Microbiol. **77**, 3202–3210 (2011). 21421777 10.1128/AEM.00133-11 PMC3126462

[36] O. Shalev , Commensal *Pseudomonas* strains facilitate protective response against pathogens in the host plant. Nat. Ecol. Evol. **6**, 383–396 (2022) 10.1038/s41559-022-01673-7. 35210578 PMC8986537

[37] S. E. Lindow, M. T. Brandl, Microbiology of the phyllosphere. Appl. Environ. Microbiol. **69**, 1875–1883 (2003). 12676659 10.1128/AEM.69.4.1875-1883.2003 PMC154815

[38] C. Vacher , The phyllosphere: Microbial jungle at the plant-climate interface. Annu. Rev. Ecol. Evol. Syst. **47**, 1–24 (2016).

[39] X.-F. Xin , Bacteria establish an aqueous living space in plants crucial for virulence. Nature **539**, 524–529 (2016). 27882964 10.1038/nature20166 PMC5135018

[40] D. S. Lundberg, S. Yourstone, P. Mieczkowski, C. D. Jones, J. L. Dangl, Practical innovations for high-throughput amplicon sequencing. Nat. Methods **10**, 999–1002 (2013). 23995388 10.1038/nmeth.2634

[41] C. R. Fitzpatrick , Chloroplast sequence variation and the efficacy of peptide nucleic acids for blocking host amplification in plant microbiome studies. Microbiome **6**, 144 (2018). 30121081 10.1186/s40168-018-0534-0 PMC6098832

[42] C. R. Fitzpatrick , Assembly and ecological function of the root microbiome across angiosperm plant species. Proc. Natl. Acad. Sci. U.S.A. **115**, E1157–E1165 (2018). 29358405 10.1073/pnas.1717617115 PMC5819437

[43] K. Vanbroekhoven , Streptomycin as a selective agent to facilitate recovery and isolation of introduced and indigenous *Sphingomonas* from environmental samples. Environ. Microbiol. **6**, 1123–1136 (2004). 15479246 10.1111/j.1462-2920.2004.00654.x

[44] B. D. Ondov , Mash: Fast genome and metagenome distance estimation using MinHash. Genome Biol. **17**, 132 (2016). 27323842 10.1186/s13059-016-0997-x PMC4915045

[45] C. Jain, L. M. Rodriguez-R, A. M. Phillippy, K. T. Konstantinidis, S. Aluru, High throughput ANI analysis of 90K prokaryotic genomes reveals clear species boundaries. Nat. Commun. **9**, 5114 (2018). 30504855 10.1038/s41467-018-07641-9 PMC6269478

[46] D. H. Parks , A complete domain-to-species taxonomy for Bacteria and Archaea. Nat. Biotechnol. **38**, 1079–1086 (2020). 32341564 10.1038/s41587-020-0501-8

[47] B. J. Callahan , High-throughput amplicon sequencing of the full-length 16S rRNA gene with single-nucleotide resolution. Nucleic Acids Res. **47**, e103 (2019). 31269198 10.1093/nar/gkz569 PMC6765137

[48] W. Ding, F. Baumdicker, R. A. Neher, panX: Pan-genome analysis and exploration. Nucleic Acids Res. **46**, e5 (2018). 29077859 10.1093/nar/gkx977 PMC5758898

[49] N. A. O’Leary , Reference sequence (RefSeq) database at NCBI: Current status, taxonomic expansion, and functional annotation. Nucleic Acids Res. **44**, D733–D745 (2016). 26553804 10.1093/nar/gkv1189 PMC4702849

[50] C. Camacho , BLAST+: Architecture and applications. BMC Bioinform. **10**, 421 (2009). 10.1186/1471-2105-10-421 PMC2803857 20003500

[51] N. Atamna-Ismaeel , Bacterial anoxygenic photosynthesis on plant leaf surfaces. Environ. Microbiol. Rep. **4**, 209–216 (2012). 23757275 10.1111/j.1758-2229.2011.00323.x

[r52] S. Siddaramappa, V. Viswanathan, S. Thiyagarajan, A. Narjala, Genomewide characterisation of the genetic diversity of carotenogenesis in bacteria of the order Sphingomonadales. Microb. Genom. **4**, e000172 (2018). 29620507 10.1099/mgen.0.000172 PMC5989583

[r53] S. Hanada, Anoxygenic photosynthesis -a photochemical reaction that does not contribute to oxygen reproduction. Microbes Environ. **31**, 1–3 (2016). 27021204 10.1264/jsme2.ME3101rh PMC4791109

[54] O. M. Finkel, T. O. Delmont, A. F. Post, S. Belkin, Metagenomic signatures of bacterial adaptation to life in the phyllosphere of a salt-secreting desert tree. Appl. Environ. Microbiol. **82**, 2854–2861 (2016). 26944845 10.1128/AEM.00483-16 PMC4836421

[55] S. J. Unterholzner, B. Poppenberger, W. Rozhon, Toxin-antitoxin systems: Biology, identification, and application. Mob. Genet. Elements **3**, e26219 (2013). 24251069 10.4161/mge.26219 PMC3827094

[56] C. Vogel, N. Bodenhausen, W. Gruissem, J. A. Vorholt, The Arabidopsis leaf transcriptome reveals distinct but also overlapping responses to colonization by phyllosphere commensals and pathogen infection with impact on plant health. New Phytol. **212**, 192–207 (2016). 27306148 10.1111/nph.14036

[57] B. Laflamme , The pan-genome effector-triggered immunity landscape of a host-pathogen interaction. Science **367**, 763–768 (2020). 32054757 10.1126/science.aax4079

[58] A. Falk , EDS1, an essential component of R gene-mediated disease resistance in Arabidopsis has homology to eukaryotic lipases. Proc. Natl. Acad. Sci. U.S.A. **96**, 3292–3297 (1999). 10077677 10.1073/pnas.96.6.3292 PMC15935

[59] D. S. Lundberg , Host-associated microbe PCR (hamPCR) enables convenient measurement of both microbial load and community composition. Elife **10**, e66186 (2021). 34292157 10.7554/eLife.66186 PMC8387020

[60] Y. Bai , Functional overlap of the Arabidopsis leaf and root microbiota. Nature **528**, 364–369 (2015). 26633631 10.1038/nature16192

[61] M. Ayliffe, C. K. Sørensen, Plant nonhost resistance: Paradigms and new environments. Curr. Opin. Plant Biol. **50**, 104–113 (2019). 31075541 10.1016/j.pbi.2019.03.011

[62] R. Panstruga, M. J. Moscou, What is the molecular basis of nonhost resistance? Mol. Plant. Microbe. Interact. **33**, 1253–1264 (2020). 32808862 10.1094/MPMI-06-20-0161-CR

[63] R. Cai , The plant pathogen *Pseudomonas* syringae pv. tomato is genetically monomorphic and under strong selection to evade tomato immunity. PLoS Pathog. **7**, e1002130 (2011). 21901088 10.1371/journal.ppat.1002130 PMC3161960

[64] K. Yoshida , The rise and fall of the Phytophthora infestans lineage that triggered the Irish potato famine. Elife **2**, e00731 (2013). 23741619 10.7554/eLife.00731 PMC3667578

[65] T. L. Karasov , The long-term maintenance of a resistance polymorphism through diffuse interactions. Nature **512**, 436–440 (2014). 25043057 10.1038/nature13439 PMC4696508

[66] F. Bansept, N. Obeng, H. Schulenburg, A. Traulsen, Modeling host-associating microbes under selection. ISME J. **15**, 3648–3656 (2021) 10.1038/s41396-021-01039-0. 34158630 PMC8630024

[67] Max Planck Institute for Biology, Contrasting patterns of microbial dominance.European Nucleotide Archive. https://www.ebi.ac.uk/ena/browser/view/PRJEB44136. Deposited 12 May 2022.

