# Supplementary material for: Contrasting patterns of microbial dominance in the Arabidopsis thaliana phyllosphere
Source: Proc Natl Acad Sci U S A. 2022 Dec 20;119(52):e2211881119. doi: 10.1073/pnas.2211881119 (PMC9907089; doi:10.1073/pnas.2211881119)
Supplement: Supplementary file 1 — Appendix 01 (PDF) [file pnas.2211881119.sapp.pdf]

Supporting Information

Materials and Methods 2
Community plant collection and lysate glycerol stock preparation 2
Collection of *Sphingomonas* isolates 2
Sphingomonas DNA isolation for short read sequencing 3
Bulk culture and additional cultured isolates 3
DNA extraction from bulk culture bacterial pellets and plant lysates 4
16S rDNA V3-V4 amplicon sequencing 4
Bacterial whole-genome sequencing and metagenome sequencing 4
Short-read genome assembly and annotation 5
Production and assembly of closed *Sphingomonas* genomes 5
Pan-genome and phylogenetic analysis 7
Construction of *Sphingomonas* BLAST database to explore gene content 7
Processing bulk culture metagenomic reads 7
Custom local reference database 8
Genome similarity comparisons 8
Inoculation, infection, and phenotyping in 24-well plates 9
Inoculation, infection, phenotyping, and hamPCR of plants on potting soil 9
Bacterial growth in whole vs. macerated leaves 10
Figure S1 12
Figure S2 13
Figure S3 14
Figure S4 15
Figure S5 16
Figure S6 17
Discussion S1 18
Figure S7 18
Discussion S2 19
Figure S8 20
Figure S9 20
Figure S10 21
Figure S11 22
Figure S12 23
Supporting Information References 24

Materials and Methods

Community plant collection and lysate glycerol stock preparation

For the community analyses, plants were harvested at Eyach (community of Starzach, 48°26'46.0" N, 8°47'02.4" E) in Germany (Fig. S1). The spring visit was on April 20, 2018, and the summer visit on September 14, 2018. For each plant species, we pooled together entire leaves from at least 6 independent plants per sample, and collected 7 such independent samples. After bringing samples back to the lab, we surface-sanitized them in 70% ethanol for 45-60 s (1), ground the fresh tissue using a sterile mortar and pestle in a volume of PBS proportional to the sample's fresh weight (7.4 mL PBS per 1 g tissue), and mixed the resulting lysate with glycerol to make -80°C freezer stocks with a final glycerol concentration of ~27%, suitable both for direct DNA extraction and culturing.

Collection of *Sphingomonas* isolates

Most *Sphingomonas* isolates (SI Dataset 3) were cultured from frozen *A. thaliana* lysates in glycerol that had originally been prepared and stored on December 11, 2015, and March 3, 2016 (2). Briefly, we processed two leaves per *A. thaliana* rosette. Each leaf was washed in 75% EtOH for 3-5 s, the ethanol was allowed to evaporate in a sterile hood, and the leaf was ground in 10 mM $MgSO_4$ before being mixed with glycerol to a final concentration of 15%-30%. The glycerol stocks were stored at -80°C. From each sample, 75 mL of lysate was plated on large Petri plates (145 x 20 mm, Greiner Bio One International, Frickenhausen, Germany) containing low-sodium Luria broth (LB, 10 g/L peptone, 5 g/L yeast extract, 1 g/L NaCl) and 1.5% agar supplemented with 100 µg/mL cycloheximide to suppress fungi (SERVA Electrophoresis, Heidelberg, Germany) and 100 µg/mL streptomycin (Thermo Fisher Scientific, Karlsruhe, Germany). Plates were incubated at room temperature for 5 to 10 d, then stored at 4°C until processing. The remaining *Sphingomonas* isolates from pooled *A. thaliana* and other plant species were recovered similarly by plating lysates on selective media (see section "Bulk culture and additional cultured isolates").

Up to 16 colonies per plate were randomly picked and transferred to 24-well plates (CELLSTAR, Greiner Bio One International) containing the same selective LB agar, and incubated for 5-10 additional d, as necessary to create a small bacterial lawn. All isolates were cultivated on agar, as we observed that many grew poorly in liquid media. Bacteria were then scraped from the agar with a sterile loop and transferred to a 2 mL screw cap tube (Starstedt, Nümbrecht, Germany) filled with 300 ml of PBS (we later switched to 300 mL of liquid LB media instead of PBS, which improved survival). Three sterile glass balls (2.85-3.45 mm or 0.25-0.5 mm diameter) (Carl Roth, Karlsruhe, Germany) were added to each screw cap to help dissociate (but not lyse) clumped bacterial cells, and tubes were shaken in a FastPrep 24 5G homogeniser (MP Biomedicals, Eschwege, Germany) at 4 m/s for 10 s. From the mixed cell suspension, 250 mL was stored at -80°C in ~27% glycerol.

Sphingomonas DNA isolation for short read sequencing

The remaining 50 mL was mixed with 150 mL of sterile PBS and transferred to a semi-skirted 96-well PCR Axygen microplates (Thermo Fisher Scientific) for DNA extraction. Bacterial cell suspensions were lysed by incubation with lysozyme (100 µg/mL final concentration) at 37°C for 30 min followed by incubation with sodium dodecyl sulfate SDS (1.5% final concentration) at 56°C for 1-2 h. Next, ⅓ volume (66 µL) of 5 M potassium acetate (CH_3COOK) was added to each well to precipitate the SDS and other cytosolic components. The 96-well plates were centrifuged at maximum speed for 20 min to pellet the precipitate, and the supernatant transferred to a new 96-well plate for genomic DNA purification using Solid Phase Reversible iImmobilization (SPRI) magnetic beads (3). Briefly, home-made SPRI bead mix adapted from (3) was thoroughly mixed with samples at a ratio of 0.6:1 bead to sample ratio and incubated for 15-20 min. The plates were placed on a magnet for 5 min and the supernatant was removed. Following two 80% EtOH washes, the beads were air-dried and resuspended in 50 µL elution buffer (EB, 10 mM Tris, pH 8.0). After an overnight incubation at 4°C, the plates were placed in the magnet and the elution buffer containing the DNA was transferred into a new 96-well PCR plate.

Bulk culture and additional cultured isolates

For *Pseudomonas* and *Sphingomonas* bulk culture analysis, approximately 50 mg of plant lysates collected from Eyach in 2018 was scooped from the frozen glycerol stocks stored at -80°C, and after thawing, 50 µL of the thawed lysate, corresponding to ~5 mg of original plant material, was pipetted onto 2% agar LB plates (200 mm diameter), using an additional 150 µL of sterile PBS to aid in spreading. This quantity of lysate was chosen empirically for our samples to avoid colonies merging into competitive lawns and to allow observation of discrete colonies. The LB medium was supplemented with 100 µg/mL cycloheximide and 100 µg/mL streptomycin for *Sphingomonas* bulk culturing, and 100 µg/mL cycloheximide and 100 µg/mL nitrofurantoin (Sigma-Aldrich, Steinheim am Albuch, Germany) for *Pseudomonas* bulk culturing (2). Plates were incubated at room temperature for 5 d for *Sphingomonas*, and for 1.5 to 2 d for *Pseudomonas*.

Individual colonies from selected plates were randomly picked to 24-well plates, as described above. To harvest the remaining bacterial colonies in bulk, plates were soaked for 5 min with 4 mL of PBS, and the surface was scraped with a flame-sterilized razor blade to loosen adherent bacteria. The plate was tilted to form a pool of PBS at the lower end, and the scraped bacteria were mixed into the PBS pool by pipetting up and down. An appropriate aliquot of the mixed suspension was then transferred to a 2 mL centrifuge tube, such that the eventual pellet would extend a maximum of 5 mm from the base of the tube (which approaches an upper limit for efficient and uniform DNA extraction using our methods).

DNA extraction from bulk culture bacterial pellets and plant lysates

For bulk culture bacterial pellets, the final pellets were resuspended in at least 750 µL of DNA lysis buffer containing 10 mM Tris pH 8.0, 10 mM EDTA, 100 mM NaCl, and 1.5% SDS. Especially large pellets (greater than 5 mm from the bottom of the tube) were suspended in proportionally greater volumes of buffer to ensure an efficient lysis, and 750 µL was used for lysis. For DNA extraction from the plant lysates in glycerol, 400 uL of glycerol lysates was mixed with 400 µL of the DNA lysis buffer described in the previous sentence, but with 3% SDS instead of 1.5% SDS to yield a final SDS concentration of 1.5%. The suspensions were pipetted to a screw cap tube containing ~0.5 mL sterile garnet rocks (Bio Spec Products Inc., Bartlesville, USA) and homogenized in a FastPrep 24 instrument at 6 m/s for 1 min. The tubes were centrifuged at 10,000 x g for 5 min, and the supernatant (about 600 µL) was mixed with 200 µL sterile 5 M potassium acetate in a 1 mL 96-well plate (Ritter Riplate 43001-0016, Schwabmünchen, Germany) to precipitate the SDS. The plates were spun at 5,000 x g for 10 min and the supernatant transferred to a new 1 mL deepwell plate. The resulting supernatant was centrifuged a second time to clear out remaining plant material and precipitate. Finally, 360 µL SPRI beads were added to 600 µL of the supernatant. After mixing and incubating on a 96-well Magnet Type A (Qiagen, Hilden, Germany), the beads were cleaned with 80% ethanol and DNA was eluted in 100 µL EB.

16S rDNA V3-V4 amplicon sequencing

The 16S rDNA V3-V4 region was amplified using the 2-step protocol described for V4 amplicons in (4), with the exception that the forward PCR primer was 341F (SI Dataset 4), and due to this different forward primer, the annealing step in the first PCR was done at 55°C instead of 50°C in (4). In addition, because the amplicons were longer than the V4 amplicons in (4), the libraries were sequenced on a MiSeq instrument (Illumina, San Diego, USA) with a V3 2 × 300 bp reagent kit instead of a V2 2 × 250 bp reagent kit. This allowed overlap and assembly of the forward and reverse reads. The frameshifts built into the primers used in the first PCR made the addition of Illumina PhiX control library to increase sequence diversity unnecessary (5), and it also enabled barcoding of more samples. This is because half of the samples from the first PCR were amplified with 341F frameshifts 1, 3, and 5, paired with 806R frameshifts 2, 4, and 6. The other half of the samples from the first PCR paired 341F frameshits 2, 4, and 6 with 806R reverse frameshifts 1, 3, and 5. The first PCR used 29 cycles and the second PCR used 6 cycles, for a total of 35 cycles. We denoised the sequences into amplicon sequence variants (ASVs) using USEARCH (6), and matched the ASVs to bacterial taxa against the RDP database (7).

Bacterial whole-genome sequencing and metagenome sequencing

DNA from each bacterial isolate, or from each metagenome, was quantified by Quant-iT PicoGreen dsDNA (Invitrogen, Waltham, USA) in a Magellan Infinite 200 PRO plate reader (Tecan Trading, Männedorf, Switzerland) and diluted and normalized to 0.5 ng/µl as a prior step to library construction. Bacterial DNA libraries were constructed using an adapted Nextera

protocol for small volumes (2, 8). In brief, 2.5 ng of DNA was sheared using Nextera Tn5 transposase (Illumina), and Nextera sequencing adapters were added though 12 cycles of PCR as previously described (2). An aliquot of each library was run in an agarose gel for quality control, and the remainder of the library was purified using SPRI beads to remove primers at a ratio of 1.5:1 beads to PCR product. The clean DNA was eluted in 40 µL of EB and the concentration of the final product was quantified with PicoGreen. Libraries were pooled in an equimolar ratio. To increase the concentration of the pool to enable further size selection procedures, the pooled library was concentrated by first precipitating the DNA by mixing with it with an equal volume of a solution containing 1 part sodium acetate and 8 parts isopropanol, passing the solution over an EconoSpin Mini Spin Columns (Epoch Life Science, Missouri City, USA), washing the column twice in 70% ethanol, and eluting in 50 µL EB. The resulting multiplexed, concentrated library molecules were size-selected to keep fragments between 350 and 700 bp using a 1.5% cassette in a BluePippin instrument (Sage Science, Beverly, USA). After adjusting the concentration of each size-selected pool to 2.5 nM, the DNA was sequenced with 2 ×150 bp paired-end reads on a HiSeq 3000 instrument (Illumina). Metadata regarding sequenced genome and metagenome samples can be found in SI Dataset 3.

Short-read genome assembly and annotation

Genomes were assembled using SPAdes genome assembler version 3.11.0 correcting for mismatches and short indels (--careful) and k-mer sizes of 21, 33, 55, 77, 99, and 127 (-k) (9). Draft genomes were corrected using Pilon version 1.20 with standard parameters (10). Annotation of bacterial genomes was accomplished using Prokka version 1.12 using the --compliant parameter (11). Coverage and N50 statistics were measured using custom scripts, including the N50.sh script from the GitHub repository of Henk den Bakker (12). The completeness of the genome was assessed using BUSCO version 2.0 (13) selecting proteobacteria lineage and the gene set (proteins) assessment (-m prot).

Production and assembly of closed *Sphingomonas* genomes

Assembly of closed *Sphingomonas* genomes required a separate DNA preparation and library production for sequencing on an Oxford Nanopore MinION instrument. First, 12 *Sphingomonas* isolates were each bulked on 15 × 15 cm^2 LB plates with 100 µg/mL streptomycin. After 2-5 days, depending on the growth rate of each strain, the bacterial lawn was harvested, and ~100 mg of bacteria was mixed with 800 µL lysis buffer, and DNA was extracted and purified using the homemade SPRI beads exactly as described above for bulk culture lysates, including the harsh bead beating. We noticed that bead beating did not shear DNA below 10 kb, and fragments greater than approximately 7 kb are sufficiently long to span repeated regions in bacterial genomes (14). The harsh bead beating during the lysis step naturally sheared the DNA to approximately 15-20 kb, and the 0.6:1 SPRI bead cleanup removed most of the smaller fragments, so no additional shearing or size selection was performed. DNA was eluted from the SPRI beads

in 400 µL EB. Because some DNA extracts remained discolored or viscous following the SPRI purification, the DNA was further purified by mixing with it with an equal volume of chloroform. The aqueous phase was collected and the HMW DNA was precipitated by mixing it with an equal volume of a solution containing 1 part sodium acetate and 8 parts isopropanol. Precipitated DNA was pelleted by centrifugation at 20,000 x g for 5 minutes. The pellet was washed twice in 70% ethanol and eluted again in 400 µL EB.

Pure DNA was prepared for sequencing on an MinION instrument (Oxford Nanopore Technologies, Oxford, UK) using the manufacturer-recommended protocol "1D Native barcoding genomic DNA" using the SQK-LSK109 kit with barcode expansions NBC104 and NBC114 for 24 samples. Briefly, DNA concentration was adjusted to 1 µg of DNA diluted in 49 µL of Ambion Nuclease-Free Water (Thermo Fisher Scientific). From this, 48 µL were treated with NEBNext reagents (NEB, Ipswich, USA) for Formalin-Fixed, Paraffin-Embedded (FFPE) samples and end repair as well as dA-tailing. The end-repaired libraries were cleaned with Agencourt AMPure XP beads (Thermo Fisher Scientific) at a 1:1 ratio and eluted in 25 µL in a 1.5 mL DNA LoBind tube (Eppendorf, Hamburg, Germany). Native barcodes were attached by ligation, the solution was again cleaned with AMPure beads at a 1:1 ratio, and concentration of all barcoded-added samples were measured using a Qubit (Thermo Fisher Scientific) fluorometer and pooled at equimolar ratios for a total of 700 ng. Finally, sequencing adapters were added to the pool by ligation. LFB was selected for washing in the final clean-up with AMPure beads. The library was prepped and loaded into an FLO-MIN106 RevD R9.4.1 flow cell following the manufacturer's instructions and sequencing was run for 24 h.

To assemble closed *Sphingomonas* genomes, Guppy package version 3.0.3 (https://nanoporetech.com/) was first used for initial base calling, to produce raw read and quality assessment in FASTQ format as recommended by (15). Next, samples were demultiplexed using qcat. Draft contigs were assembled *de novo* by using mini_assemble, which is part of the Pomoxis toolkit (version 0.3.6, https://github.com/nanoporetech/pomoxis), using default parameters for de-novo assembly with miniasm (16) and four rounds of long-reads-based polishing with minimap2 and Racon. Each of the genomic consensus assemblies was further improved by four additional rounds of long-read-based polishing with Racon followed by one additional round using medaka_consensus (package version 0.6.5, https://github.com/nanoporetech/medaka). To enhance base corrections, an additional polishing step using high-quality Illumina short reads from the same *Sphingomonas* strains was used. First, the short reads were mapped onto the assembled *Sphingomonas* genome, using Burrows-Wheeler Alignment (BWA) with BWA-MEM (17). Next, these mapped reads were used to correct the assembly using Pilon version 1.23 with default parameters (10). Assessment of genome completeness and annotations were performed in the same way as short-read draft genomes. Metadata regarding sequenced closed genomes can be found in SI Dataset 3.

Pan-genome and phylogenetic analysis

The panX pan-genome pipeline (18) was used to assign orthology clusters and construct the phylogenetic tree, taking as input the Genbank format files (.gbk) from Prokka (previous section). The following parameters were used for the panX analysis: the divide-and-conquer algorithm (--dmdc), a size of 50 strains per subset to run DIAMOND (-dcs 50) (19), and a soft core genome cutoff of 70% that includes all genes present in >70% of the strains as part of the core genome (-cg 0.7). Genomes included in each panX run are indicated in SI Dataset 3.

Sphingomonas BLAST database to explore gene content

A list of genes hypothesized to be important for *Sphingomonas* biotic interactions was assembled (SI Dataset 1), and all available *Sphingomonas* predicted protein sequences from these genes were downloaded from NCBI RefSeq, with manual removal of obviously truncated or low-quality sequences. Because many sequences were highly similar, the sequences were first clustered with USEARCH (6) to produce Operational Taxonomic Units (OTUs) of 95% sequence identity, and the representative sequences of these OTUs were then used to create a BLASTP (20) database that incorporated known allelic variation in the various genes. To align the *Sphingomonas* genomes, we predicted translated proteins for all genes from the genomic FASTA sequences using AUGUSTUS-3.3 (21), and aligned the translated protein sequences to the BLASTP database using BLAST-2.9.0+. We filtered the BLASTP results to those with at least a 30% identity query to reference sequence threshold, a 60% alignment length threshold of query to reference sequence and a 60% length ratio threshold of query and reference sequence.

Identification of known plasmids in draft genomes

Bacterial genomes were first segmented into non-overlapping 2000 bp pieces using the “fold” command in linux, and each fragment was aligned to the 16 circularized plasmid sequences from our 12 complete genomes using Minimap2 (57) using default parameters. We removed low quality alignments with fewer than 200 bases mapping and with quality scores less than 20, and then counted the total number of high-quality bases aligned to each plasmid. In Fig. 2f, plasmids needed to be covered over at least 30% of their total length to show up on the heatmap.

Processing bulk culture metagenomic reads

A first quality control of all raw metagenome sequencing data was performed using Skewer version 0.2.2 (22) to trim raw reads and to remove highly degenerative reads (-n) or reads shorter than 20 bp (-l 20).

Mapping with BWA-MEM. For samples with a total yield of at least 500 Mb, the filtered reads were mapped with BWA-MEM (17) using standard parameters against a custom-made reference database (described in the next section). Reads mapping with a quality score of 30 or higher were output to a BAM file using SAMtools (23). Duplicated reads were removed using the

MarkDuplicates command in Picard tools version 2.0.1 (http://broadinstitute.github.io/picard/) using default parameters. SAMtools -stats and -fastq commands were used to retrieve BWA-MEM mapping statistics and convert the BAM files back to FASTQ files, respectively (SI Appendix Fig. S10). The mapping results with BWA-MEM are shown in Fig. 5.

Mapping with DiTASiC. A concern when mapping metagenomic reads to bacterial reference genomes is that some reads may map equally well to multiple references. DiTASiC (24) is specifically designed to overcome problems with these shared reads and accurately infer the abundance of each strain. The first step in the process is creating a similarity matrix of reference genomes using "ditasic_matrix.py". For this, the read length used (-l) was 150 bp, and to reduce computation time, the number of simulated reads sampled per reference genome (-n) was reduced from 250,000 to 100,000. Subsequent steps were performed using default parameters. Abundance estimates produced with DiTASiC are shown juxtaposed with corresponding BWA-MEM results in SI Appendix Fig. S12

Custom local reference database

Metagenome reads from the *Sphingomonas* or *Pseudomonas* bulk cultures were mapped to a three part custom-made reference database of bacterial genomes. First, "Decoy" genomes contained bacteria from other genera, and were used to help classify reads from contaminant bacteria. The collection of Decoy genomes was produced by downloading the *A. thaliana* phyllosphere, root, and soil isolates from (25) and removing *Pseudomonas* when mapping *Sphingomonas,* or removing *Sphingomonas* when mapping *Pseudomonas.* Three different genome sets were combined with distinctive headings to allow later recovery of mapped reads:

DECOY: A set of bacterial genomes (~430 genomes) found in *A. thaliana* phyllosphere, roots, and soil surrounding the plants. Published in (25). Decoy genomes had the mission to capture and identify contaminant reads.
REFSEQ: A set of bacterial genomes of *Pseudomonas* or *Sphingomonas* found in the NCBI Reference Sequence Database (26).
LOCAL: A set of bacterial genomes of interest sequenced in-house. This included 165 genomes representative of local *Pseudomonas* isolates sequenced by (2) and all the single *Sphingomonas* and *Pseudomonas* genomes from this study.

Genome similarity comparisons

Briefly, to generate similarity matrices for *Sphingomonas* and/or *Pseudomonas* genome comparisons using MASH (version 2.1) (27), we used the formula 100 × (1-MD), where MD is the MASH distance, to convert MASH distances to similarity scores. To calculate average nucleotide identity (ANI), we used FastANI (28) For similarity matrices Heatmaps of similarity scores were illustrated using the function "heatmap.2" in the R package "gplots" (29).

Inoculation, infection, and phenotyping in 24-well plates

Plant cultivation: Each well of a 24-well plate (Greiner) was filled with 1.5 mL of 1% agar (Duchefa Farma, Haarland, Netherlands) containing half-strength Murashige-Skoog (MS) medium with MES buffer. *Arabidopsis thaliana* seeds (accession Ey15-2, CS76309) were surface-sterilized by submergence in 70% EtOH with 0.01% Triton X-100 for 1 min, then submerging in 10% household bleach solution for 12 min, and finally washing three times with sterile water. The seeds were then stratified at 4°C for 3 d in water, and then were pipetted onto the agar (1 seed/well). Excess water after distributing seeds was removed by pipetting.

Inoculation and infection: Freshly-sown, ungerminated seeds were inoculated with 4 µL of 10 mM $MgCl_2$ or with *Sphingomonas* sp. suspended in the same amount of buffer to an optical density at 600 nm (OD_{600nm}) of 0.5. The *Sphingomonas* colonies, which had been cultivated for 5 d on selective LB agar plates with 100 µg/mL streptomycin, were scraped from the plates with a sterile loop, and were washed twice by centrifugation and resuspension in $MgCl_2$ to remove residual antibiotics. Inoculated seeds were germinated and seedlings were cultivated for 10 d in growth chambers at 21°C with 16 h of light. The seedlings were then challenged with 100 µL of 10 mM $MgCl_2$ or with *Pseudomonas* suspended in the same amount of buffer to OD_{600nm} = 0.01. The bacteria were drip-inoculated by pipette to the center of each rosette. Plates were sealed with Parafilm and returned to the growth chamber for 7 d.

Plant phenotyping: On 0, 2, 4, and 7 d post inoculation, rosettes were imaged in the plates with a custom procedure to eliminate glare. Briefly, an opaque box was filled with a LED light source and covered with a sturdy translucent paper surface to diffuse the light. Each plate was placed on top of the paper in a defined position, and the backlighting allowed imaging from above without removing the lids. Pictures were taken using a LUMIX DMC-TZ71 digital camera (Panasonic, Osaka, Japan) without flash. Images were processed similarly to what has been described (30). Briefly, a predefined mask was used to extract each plant in the image, and automatic segmentation based on pixel color was applied to recognize plant leaves from background. The leaf area of each plant was then calculated based on the segmented plant images.

Inoculation, infection, phenotyping, and hamPCR of plants on potting soil

Plant cultivation: *Arabidopsis thaliana* seeds were surface-sterilized, stratified for 4 days in sterile water at 4°C, and sowed on potting soil (CL T Topferde; www.einheitserde.de) in 7 cm pots (PL 2832/20, Pöppelmann, Lohne, Germany). Seedlings were germinated and cultivated under short day (8 h light) growing conditions at 23°C and 65% relative humidity, illuminated by cool white fluorescent light of 125 to 175 µmol m^{-2} s^{-1}.

Inoculation and infection: Two weeks after sowing, seedling leaves were sprayed ad- and ab-axially with *Sphingomonas* strains cultivated identically as for inoculation in 24-well plates described above, but resuspended in 10 mM $MgCl_2$ buffer to a concentration of OD_{600nm} = 1.0.

Plants were also sprayed with heat-killed (boiled) *Sphingomonas* prepared by mixing equal parts of all strains after they had been resuspended at OD_{600nm} = 1.0 in $MgCl_2$ and boiling the resulting solution for 10 minutes. Humidity domes were kept on the flats for 48 h. On the fourth day (after 96 h) following *Sphingomonas* treatment, plants were sprayed with *Pseudomonas* strains also cultivated identically as for 24-well plates, but resuspended in 10 mM $MgCl_2$ buffer to a concentration of OD_{600nm} = 1.0. A heat-killed *Pseudomonas* mix was also prepared, as described above. Prior to spray-inoculating *Pseudomonas,* the surfactant Silwet L-77 added at 0.04% v/v following the protocol in (31). Humidity domes were kept on the flats for 72 h.

Plant phenotyping: Five days (120 h) post inoculation, overhead images of pots arranged in the flat were taken from a height of ~ 1.5 m with a LUMIX DMC-TZ71 digital camera. Because the position of each pot in the flat and the position of each plant in the pot was not fixed in the overhead images, identically-sized squares containing each plant were manually cropped from each image and arranged into a montage for each flat using object alignment functions in Illustrator (Adobe, Mountain View, USA). The montages were processed as described in the previous section by using a predefined mask to extract each plant in the image and counting green pixels (SI Dataset 2).

Confirmation of viable bacteria and DNA extraction: Following plant phenotyping, whole seedlings were harvested into 2 mL screw-cap tubes (Type I, Sarstedt, Nümbrecht, Germany) using flame-sterilized tweezers and scissors, and kept on ice. First, one 5-mm glass bead (Sigma, St. Louis, USA) and 300 µL of PBS buffer were added to the tubes and the tubes were shaken at 4 m/s in a FastPrep 24 for 20 sec to release viable bacteria from the leaves. From this homogenate, 20 µL was directly plated on both *Sphingomonas* and *Pseudomonas* selective media to confirm that viable bacteria were present at the end of the experiment. Next, 470 µL of DNA lysis buffer containing 3% SDS as described in section "DNA extraction from bulk cultures and plant lysates" above was added to the remaining lysate for a final SDS concentration of 1.88%. Garnet rocks (0.5 mL) were added to the lysate and DNA was extracted by bead-beating at 6 m/s in the FastPrep 24, and purified as described above.

hamPCR to determine bacterial load and composition the bacterial community: hamPCR (32) was performed on seedlings from soil-grown plants using primers for the *A. thaliana GIGANTEA* gene as a host gene and primers for the V4 region of the 16S rDNA (SI Dataset 4), using primers and cycling conditions recommended in (32). Metadata regarding sequenced hamPCR amplicons can be found in SI Dataset 3.

Bacterial growth in whole vs. macerated leaves

Sample preparation: In spring 2021, ~40 g each of wild *A. thaliana* and *Brassica napus* leaves were collected from a local field site in Germany (community of Kusterdingen, 48°31'00.9" N, 9°06'34.9" E) with sterile scissors and tweezers, and kept cool on ice. The larger *B. napus* leaves

were trimmed into pieces no larger than ~5 cm^2. Upon returning to lab, the *A. thaliana* and *Brassica napus* leaves were washed in separate batches with copious amounts of distilled water and finally autoclaved sterile water to remove as many dirt and sediment-associated microbes as possible. From each species, 9 large aliquots (~2 g) and 24 small aliquots (~0.5 g) were prepared. One of the 9 large aliquots was macerated, diluted in an equal weight of PBS, and used for culturing bacteria on LB media. The other 8 large aliquots, to be used for repeated sampling of the macerated bacterial population, were each ground in a sterile mortar and pestle and each macerate was transferred to an empty petri plate. The 24 small aliquots were each placed with leaves adaxial (upper) sides up in a separate petri plate. All macerated leaves, whole leaves, and plated bacteria were incubated in 16°C in 16 h of light.

For sampling on each of 0, 1, and 2 d, eight of the small aliquots were sacrificed and ground in a mortar and pestle, and ~0.3 g of each resulting homogenized sample was transferred to a pre-weighed screw-cap tube already containing 400 µL PBS and two 5-mm glass balls for weighing, CFU-counting, and 16S rDNA sequencing. Likewise, ~0.3 g from the already macerated large lysate was removed using a sterile steel spoon and transferred to a pre-weighed screw-cap tube for the same procedures. At 2 d, bacteria that had grown from the macerates that had been plated on LB plates were also collected.

<u>CFU counting:</u> Each screen cap tube containing 400 µL was ground for 20 seconds at speed 4.0 m/s in a FastPrep 24 to release viable bacteria from the leaves. Then 400 µL of additional PBS was added and 20 µL was plated in a dilution series on LB media with 100 µg/mL cycloheximide and 100 µg/mL nitrofurantoin to select *Pseudomonas*.

<u>16S rDNA sequencing:</u> After the fresh lysate had been removed for CFU counting, 65 µL of 20% SDS (~1.625 % final) and 0.5 mL garnet rocks were added. This was processed as described in the section "DNA extraction from bulk culture bacterial pellets and plant lysates". The resulting DNA was amplified with V4 rDNA primers and sequenced as described in the section "16S rDNA V3-V4 amplicon sequencing", with the exception that the forward primer was 515F as in (4), the annealing step was done at 50°C, and the material was sequenced using paired 2×150 HiSeq 3000 reads. This sequencer was chosen because the experiment was small and it was expedient to spike the libraries into a lane of unrelated material. Because the 150 bp reads could not reliably be assembled into full V4 amplicons and a single 150 bp read alone could not provide sufficient resolution to distinguish SphASV1 and PseASV1 from other bacteria in the genus, the forward and reverse reads were simply concatenated and the concatenated sequences corresponding to PseASV1 and SphASV1 were identified and quantified separately. All other sequences were classified to the level of bacterial families based on the forward read alone. Metadata regarding these sequenced amplicons can be found in SI Dataset 3.

Figure S1

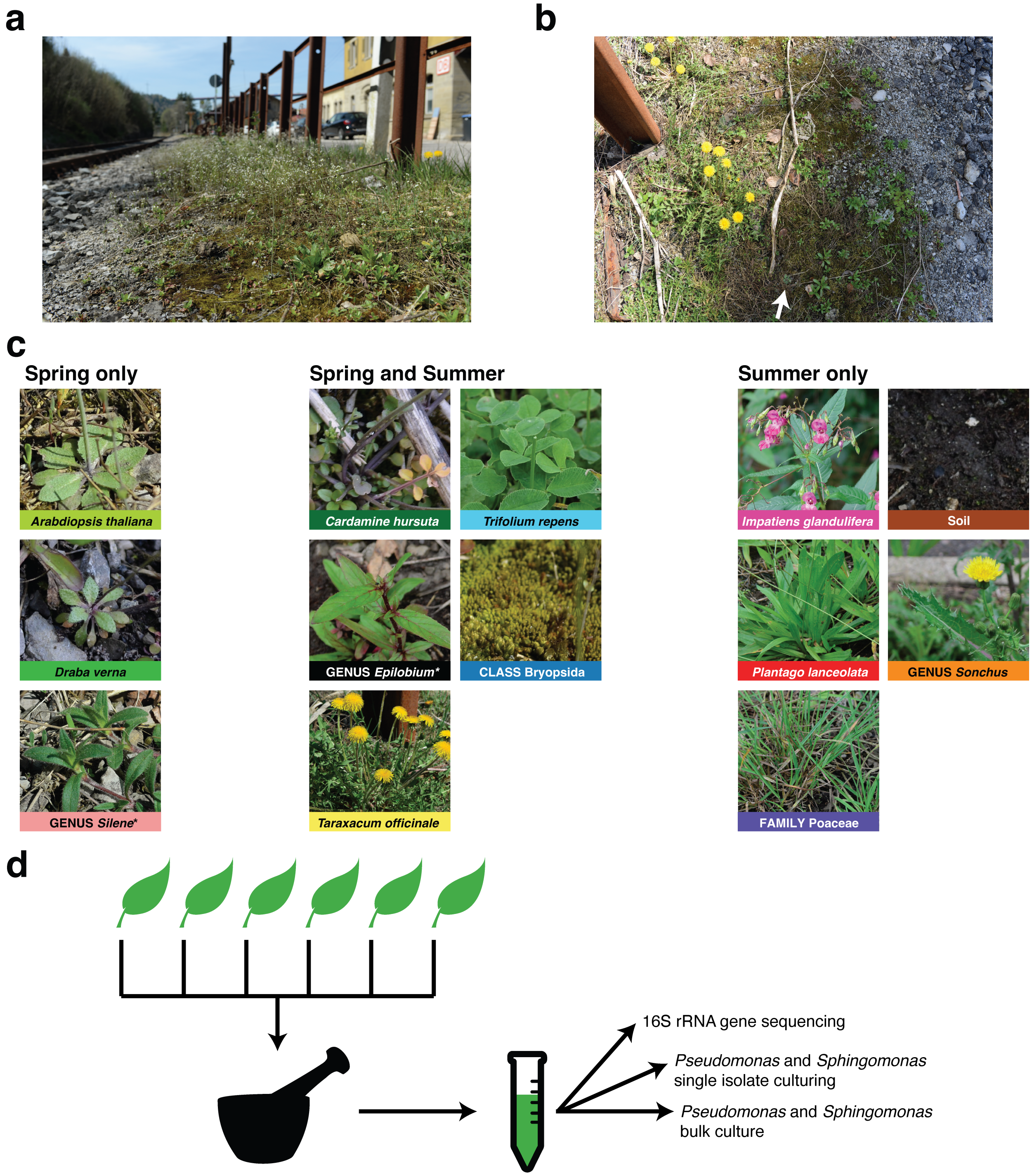

Fig. S1 | Sampling scheme. a, The harvested area of Eyach, Germany on 20 April, 2018, (spring), viewed from the ground. The small white flowers are predominantly from *A. thaliana*. **b**, Part of the harvested area from **(a)** viewed from above, showing *A. thaliana* (arrow) and surrounding plants. **c**, Common plant species at the site were harvested only in spring (left), both spring and summer (September 14, 2018, middle), and only summer (right). Soil was only harvested in summer. If the plant species could be identified, the full scientific name is given. For all other plants, a higher taxonomic level is given. The names with asterisks (*) represent less confidence in the species designation, as they are estimates based on classification of chloroplast rDNA sequences. **d,** For each plant species, at least 1 leaf from at least 6 individuals per species was pooled to make a single sample. Seven such samples were

produced per plant species. The leaves were ground in PBS in a mortar and pestle, and the macerate was mixed with glycerol to make a cryo-protected -80°C freezer stock to be used both for nucleic acid extraction and for culturing live bacteria.

Figure S2

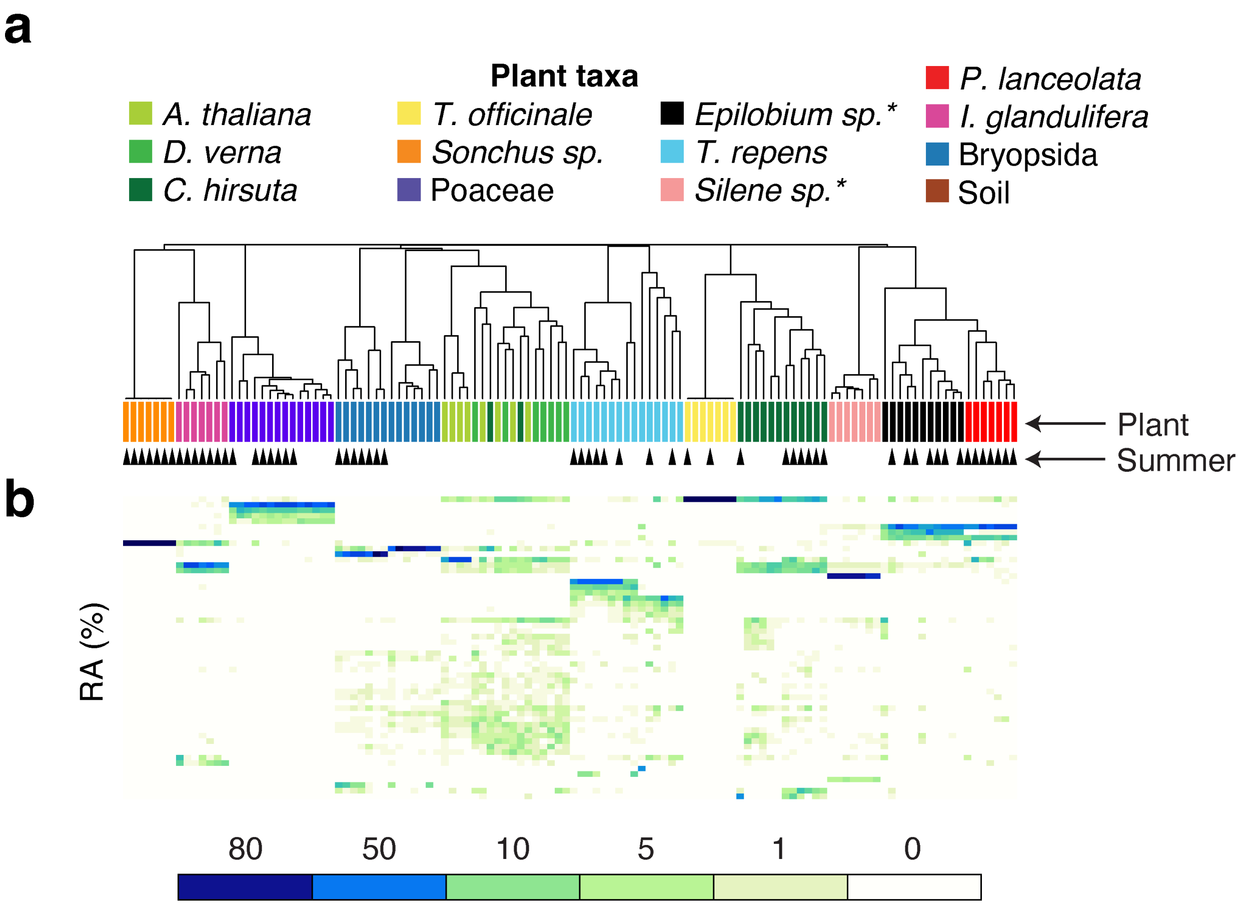

Fig. S2 | Analysis of sampled plant chloroplast sequences. a, The relative abundances of 16S rDNA sequences classified as "chloroplast" were used to calculate pairwise Bray Curtis dissimilarity scores for all samples with ≥100 chloroplast sequences; the dissimilarity matrix was then used to cluster plants. **b,** Heatmap of chloroplast ASV relative abundances (RA%), showing the top 100 most abundant sequences. The tight clustering by plant species reveals that our identification of different plant taxa by their visual phenotype corresponded well with their genetics.

Figure S3

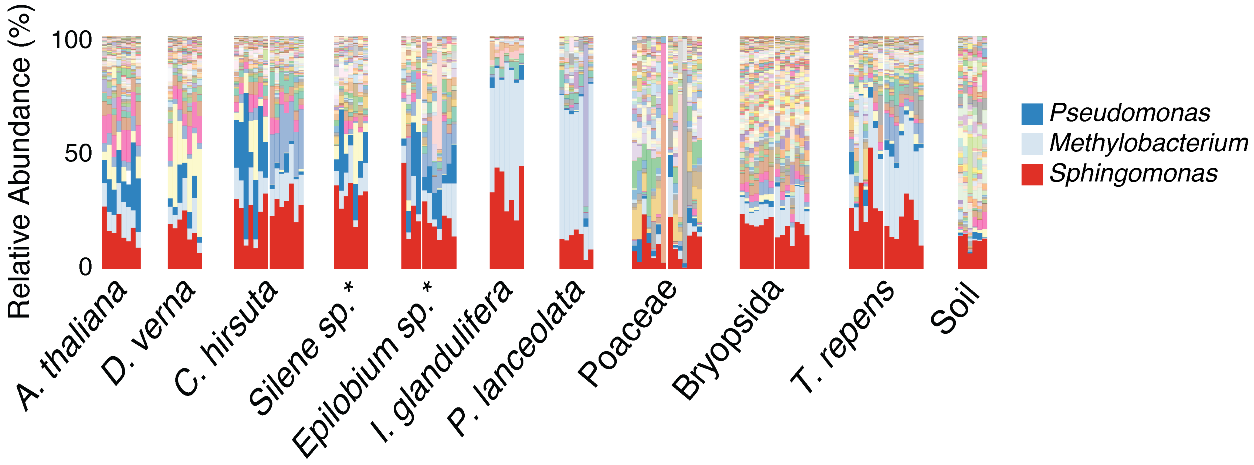

Fig. S3 | Relative abundance of major bacterial genera across plant species. Stacked bars represent the relative abundances (RA%) of bacterial genera in the present study (in contrast to the bacterial families shown in Fig. 1) with the top 3 bacterial genera shown in reverse order in the legend at right. In contrast to Fig. 1, samples are grouped by plant species and not hierarchically clustered. For plants present in spring and summer samplings (*C. hirsuta*, *Epilobium sp.*, Poaceae, Bryopsida, *T. repens*), spring samples are grouped to the left and summer to the right.

Figure S4

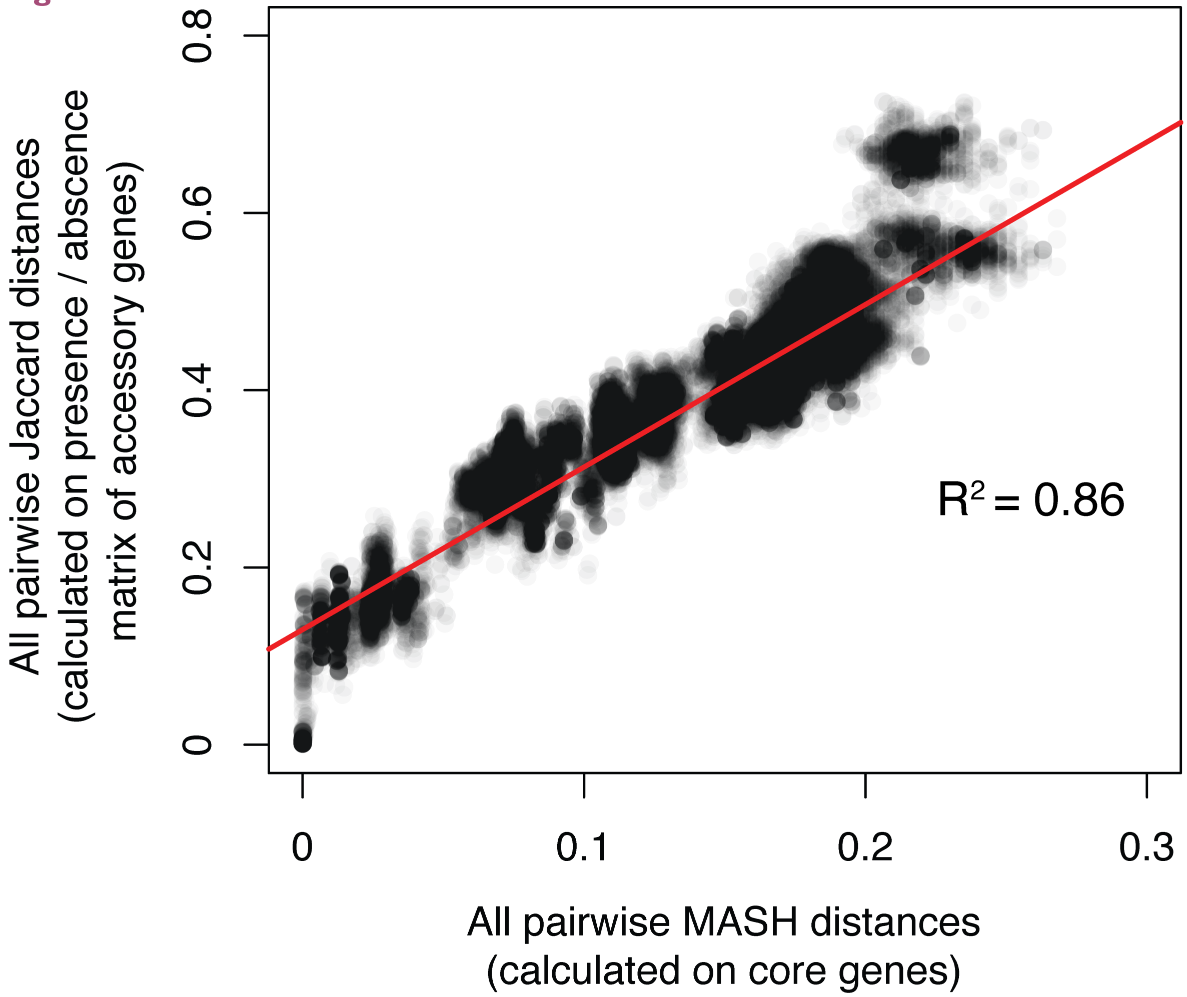

Fig. S4 | Correlation of gene presence or absence in the accessory genome with differences in the core genome. The presence/absence matrix of accessory genes (Fig. 2e) was used to calculate pairwise Jaccard distance between *Sphingomonas* strains. Each pairwise distance was plotted against the corresponding pairwise MASH distances (27) calculated on the core genomes.

Figure S5

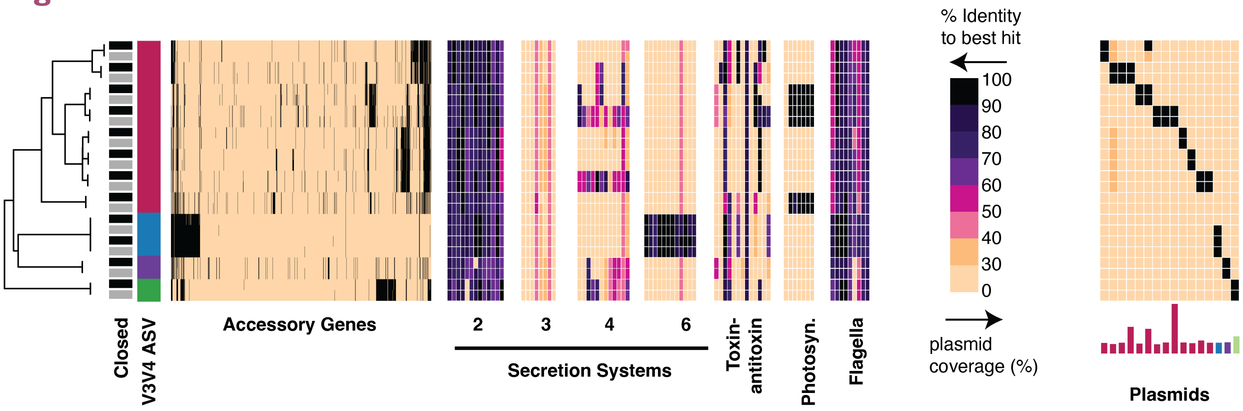

Fig. S5 | Closed genomes compared to their draft counterparts. Nanopore-sequenced closed genomes (black boxes next to tree) are neighbors with their corresponding Illumina draft genomes (grey boxes next to tree) in the core genome maximum-likelihood tree, and share the same gene presence and absence patterns with few exceptions. Most notably, one of the draft genomes has full coverage of a plasmid identified in other closed genomes, while the corresponding closed genome misses those genes, suggesting that perhaps in re-cultivation of the stock for Nanopore sequencing this plasmid was lost. The order of genomes (first Nanopore, then Illumina) from top to bottom is: S216H113, S133H113, S127H113, S18H113, S213H113, S190H113, S230H113, S237H113, S380H113, S136H113, S132H113, and S337H113.

Figure S6

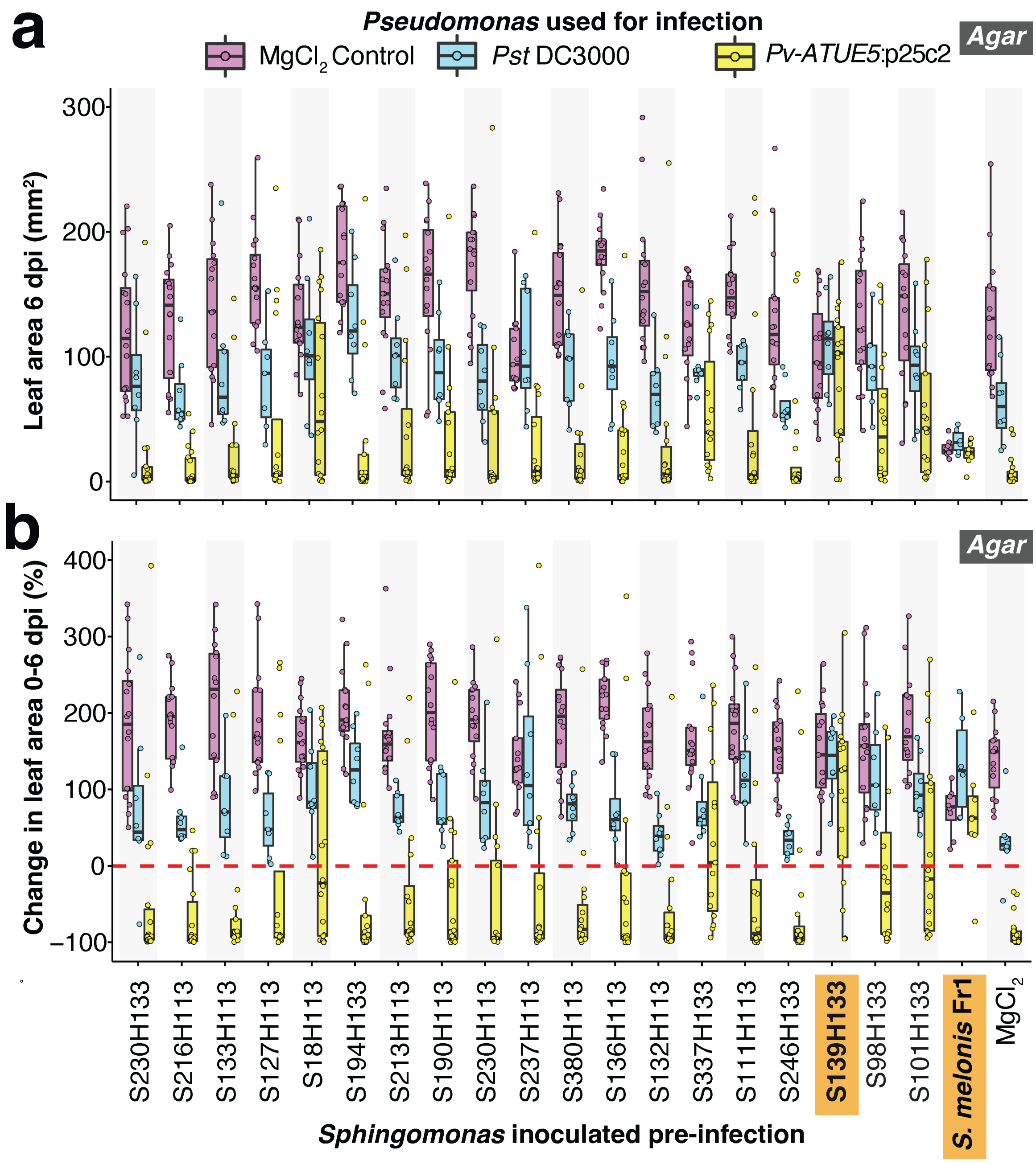

Fig. S6 | Gnotobiotic plant protection experiment for all tested strains. a-b, Boxes enclose the interquartile range (IQR) with whiskers extending to up to 1.5 times the IQR. **a**, In an independent experiment from that shown in Fig. 3a, 19 local *Sphingomonas* isolates, *S. melonis* Fr1, or $MgCl_2$ buffer were used to pre-treat *A. thaliana* Ey15-2 seeds germinating in 24-well agar plates, and on day 10 seedlings were challenged with a $MgCl_2$ control, *Pto* DC3000, or *Pv-ATUE5*:p25c2 and monitored for 6 dpi. The Y-axis shows the rosette size at 6 dpi, with the dotted red horizontal line representing no change. **b**, Percent change in rosette size between 0-6 dpi. The *Sphingomonas* highlighted in orange showed significant protection with no difference in symptoms from *Pst* DC3000 across this replicate and the replicate shown in Fig. 3a (FDR-adjusted Mann-Whitney U-test, $p > 0.05$).

Discussion S1

We also observed a stunting phenotype of *S. melonis* Fr1 in the Col-0 accession (not shown), for which a protective effect of *S. melonis* Fr1 had been previously reported (33). Although the authors did not observe any negative effects of protective *S. melonis* Fr1 on plant growth (34), they did report that the plant transcriptomic response to the strain involved induction of a set of defense genes overlapping those induced by *Pst* DC3000 (33, 34). We therefore suspect that the unexpected plant stunting caused by *S. melonis* Fr1 in our experiments might have been due to differences in our gnotobiotic system or in the growth media that enhanced plant immune system activation by *S. melonis* Fr1 to the point where it overstimulated the plants, thus causing growth defects.

Figure S7

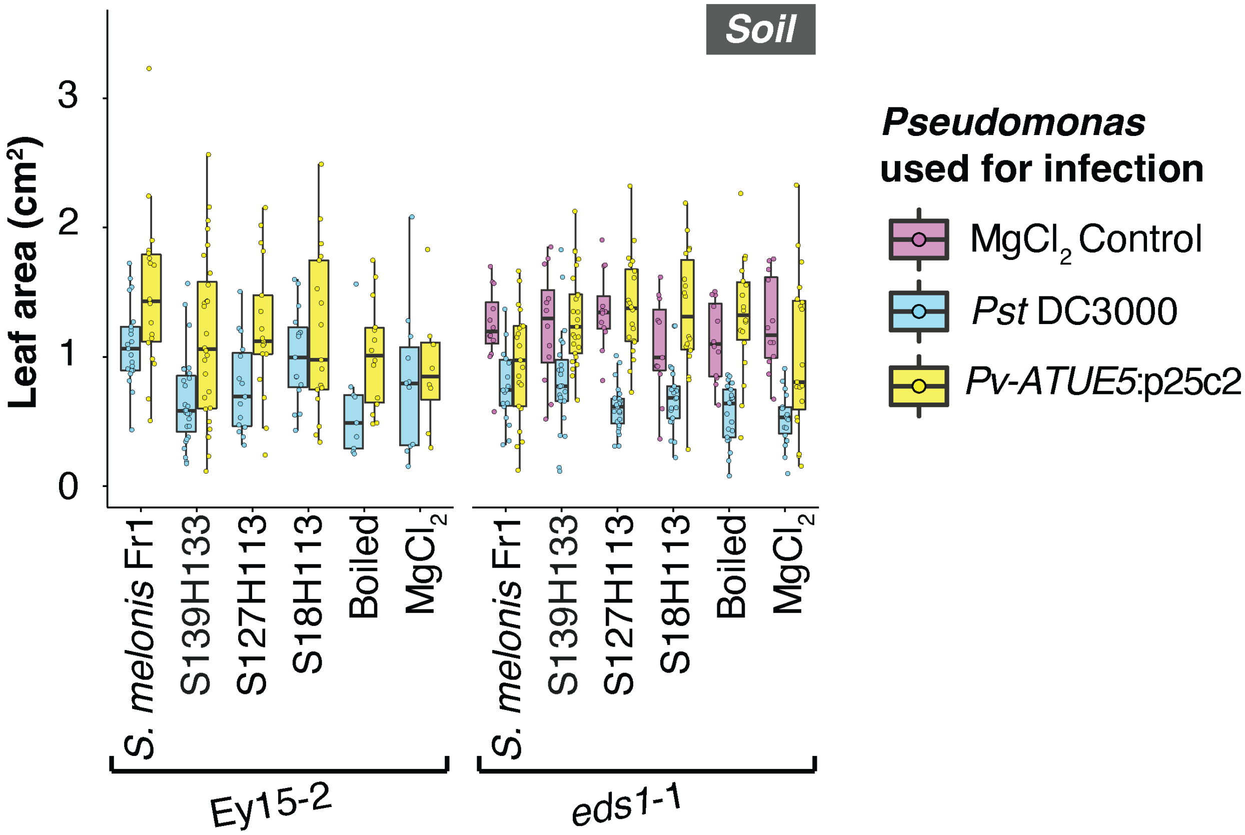

Fig. S7 | Absence of *Sphingomonas* protection against *Pst* DC3000 on soil. Absolute sizes of rosettes of Ey15-2 and Col-0 *eds1*-1 plants grown on soil and treated with different *Sphingomonas* and *Pseudomonas* combinations, elaborated from Fig. 3d. The $MgCl_2$ control spray for *Pseudomonas* was omitted for the Ey15-2 genotype due to an insufficient number of available plants. Unlike on agar, *S. melonis* Fr1 treatment did not stunt the growth of seedlings. Plants not pre-treated with were larger than non-treated (buffer) plants for any genotype or *Pseudomonas treatment.* Boxes enclose the interquartile range (IQR) with whiskers extending to up to 1.5 times the IQR.

Discussion S2

The unexpectedly weak *Pv-ATUE5*:p25c2 virulence prompted us to additionally test the *A. thaliana* mutant *coronatine-insensitive 1* (*coi1*-16), which is more susceptible to *P. viridiflava* (35) due to defects in defense responses mediated by jasmonic acid (JA). We therefore infected both *Pv-ATUE5*:p25c2 and *Pst* DC3000 on *coi1*-16 (Col-0 background), *eds1*-12 (Col-0 background) (36), and wild-type Col-0 using the same strong infection conditions.

We observed no obvious discoloration or disease symptoms on any plant, although *Pv-ATUE5*:p25c2 did significantly retard the growth of rosettes compared to a 10 mM $MgCl_2$ control in all genetic backgrounds (Mann-Whitney U-tests with FDR adjustment, $p < 0.01$), in agreement with reports of *P. viridiflava* virulence on soil (37, 38), and the magnitude of this effect was strongest in the *coi1*-16 mutant (SI Appendix Fig. S8), consistent with JA increasing resistance to *P. viridiflava* (35). In contrast, *Pst* DC3000 induced strong chlorosis on both Col-0 and *eds1*-1, but not on *coi1*-16, and retarded the growth of all plants, with *coi1*-16 being the least affected (SI Appendix Fig. S8), consistent with the *coi1*-16 mutant being more resistant to *Pst* DC3000 (39). In addition to scoring disease symptoms and plant growth, we estimated *Pseudomonas* load in the mutants using hamPCR (40), a quantitative amplicon sequencing approach that derives bacterial load by relating abundance of 16S rDNA to a single-copy host gene. For Col-0 and *eds1*-1 plants, the load of *Pst* DC3000 exceeded the limits of robust quantification, while it was much lower on resistant *coi1*-16 plants, as expected. *Pv-ATUE5*:p25c2 loads were far lower than *Pst* DC3000 on all plants, explaining the milder virulence phenotype.

Figure S8

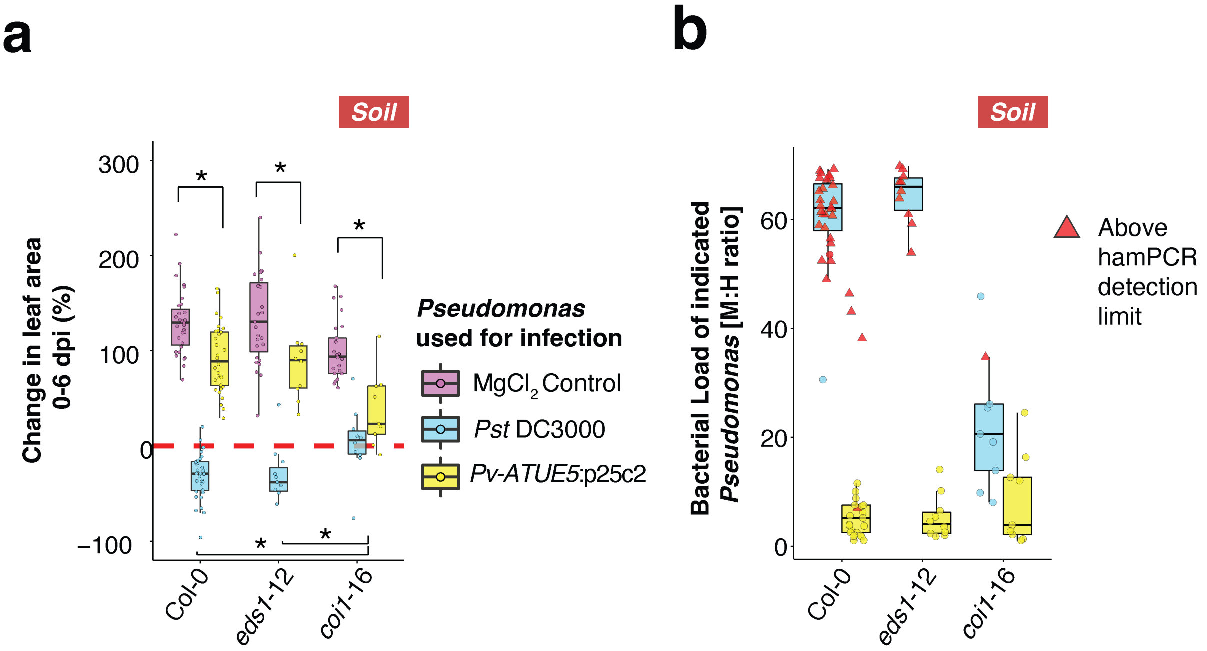

Fig. S8 | *Pseudomonas* virulence in *A. thaliana* Col-0 plants on soil. a-b, Boxes enclose the interquartile range (IQR) with whiskers extending to up to 1.5 times the IQR. **a**, Percent change in rosette size between 0-6 dpi for three genotypes in the Col-0 background infected with *Pseudomonas* or buffer control and grown on soil. **b**, Bacterial load ratio in plants infected with *Pst* DC3000 as determined by hamPCR. Note that the color legend represents bacteria quantified, not the bacteria used for treatment. *Pseudomonas* isolates are indicated by the color legend in panel **(a)**.

Figure S9

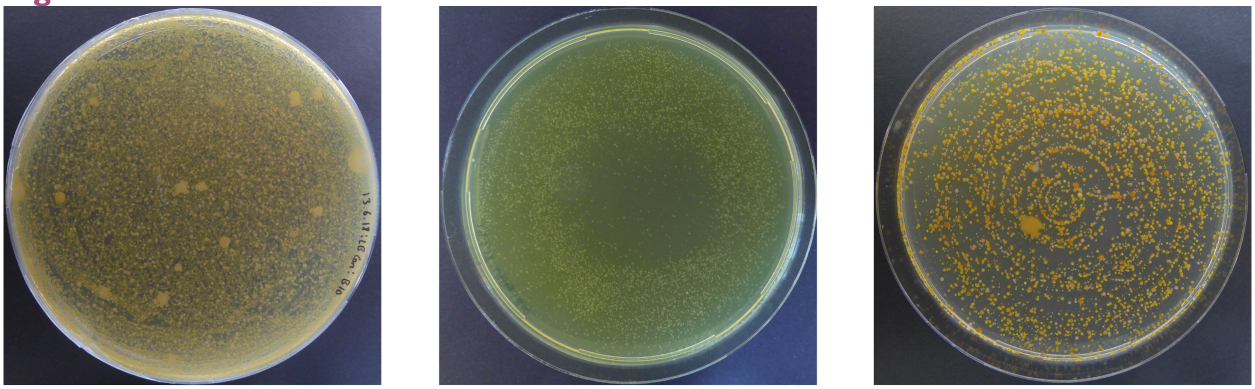

Control
(LB + cycloheximide)

Pseudomonas selection
(LB + cycloheximide + nitrofurantoin)

Sphingomonas selection
(LB + cycloheximide + streptomycin)

Fig. S9 | Control and selective media plates for bulk culture of *Pseudomonas* and *Sphingomonas*. From each lysate, 50 µL of glycerol stock corresponding to ~5 mg of original plant material was plated on selective *Pseudomonas* or *Sphingomonas* media. All three plates represent the same plant sample (*D. verna*, HOST_PLANT_ID = "Spring10")

Figure S10

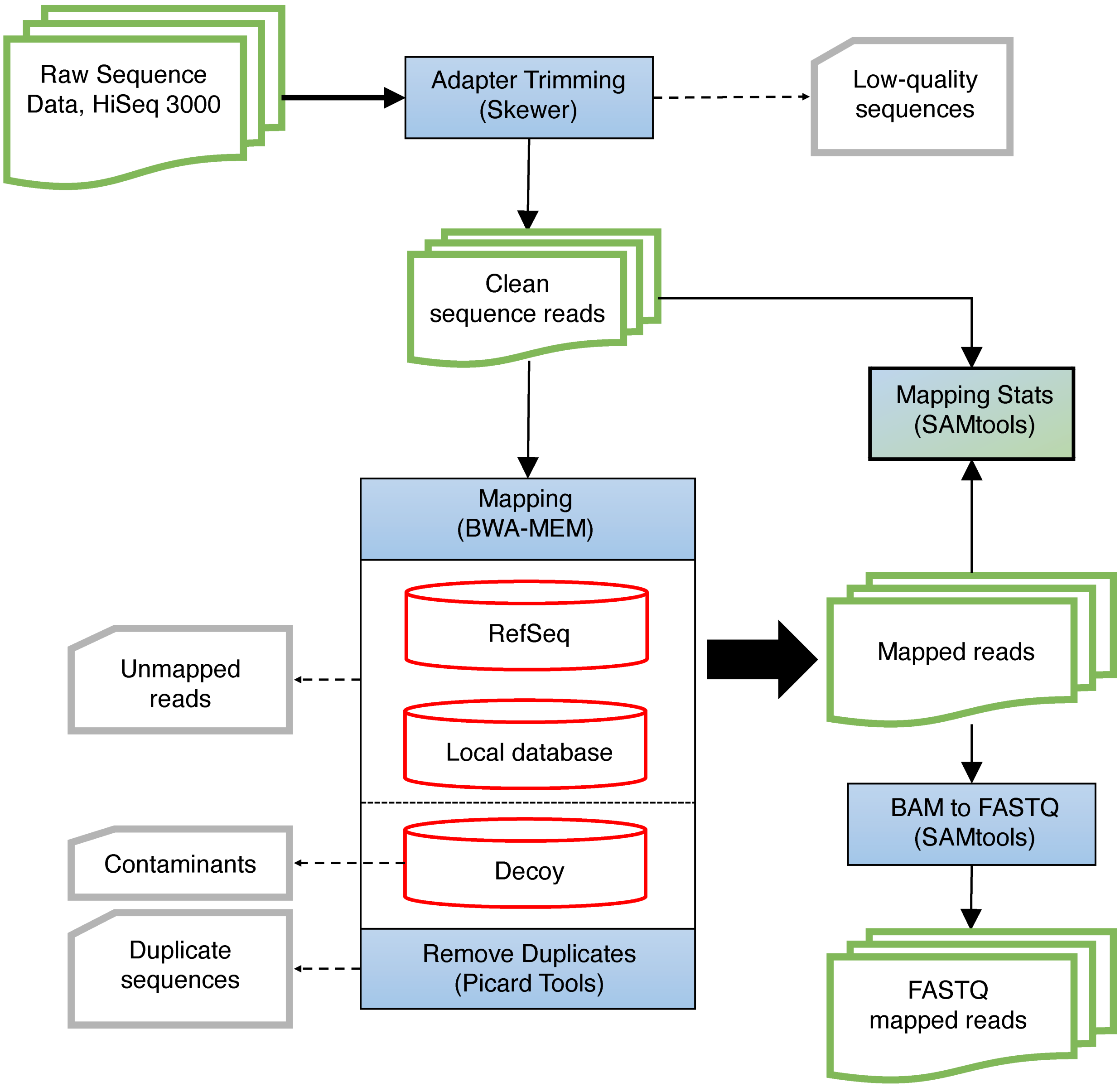

Fig. S10 | Metagenome analysis pipeline used in this study. Raw sequences were trimmed, filtered, and mapped to a reference genome. The reads of interest were outputted as FASTQ files for further analysis.

Figure S11

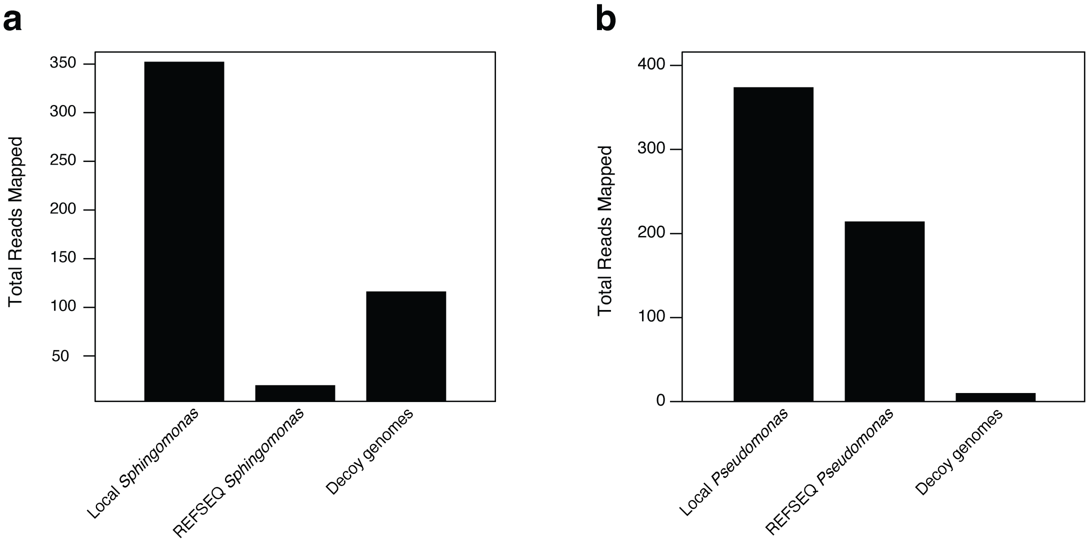

Fig. S11 | Metagenome reads mapping to LOCAL, REFSEQ, and DECOY genomes. a, All bulk culture *Sphingomonas* metagenomes were mapped to local *Sphingomonas* genomes (left), *Sphingomonas* genomes from NCBI's REFSEQ (center) and Decoy plant associated genomes from other genera to capture contaminants (right). Decoy reads represented 30% of all mapped reads. **b**, Same as **(a)**, but for *Pseudomonas*. Decoy reads represented 1.7% of all mapped reads.

Figure S12

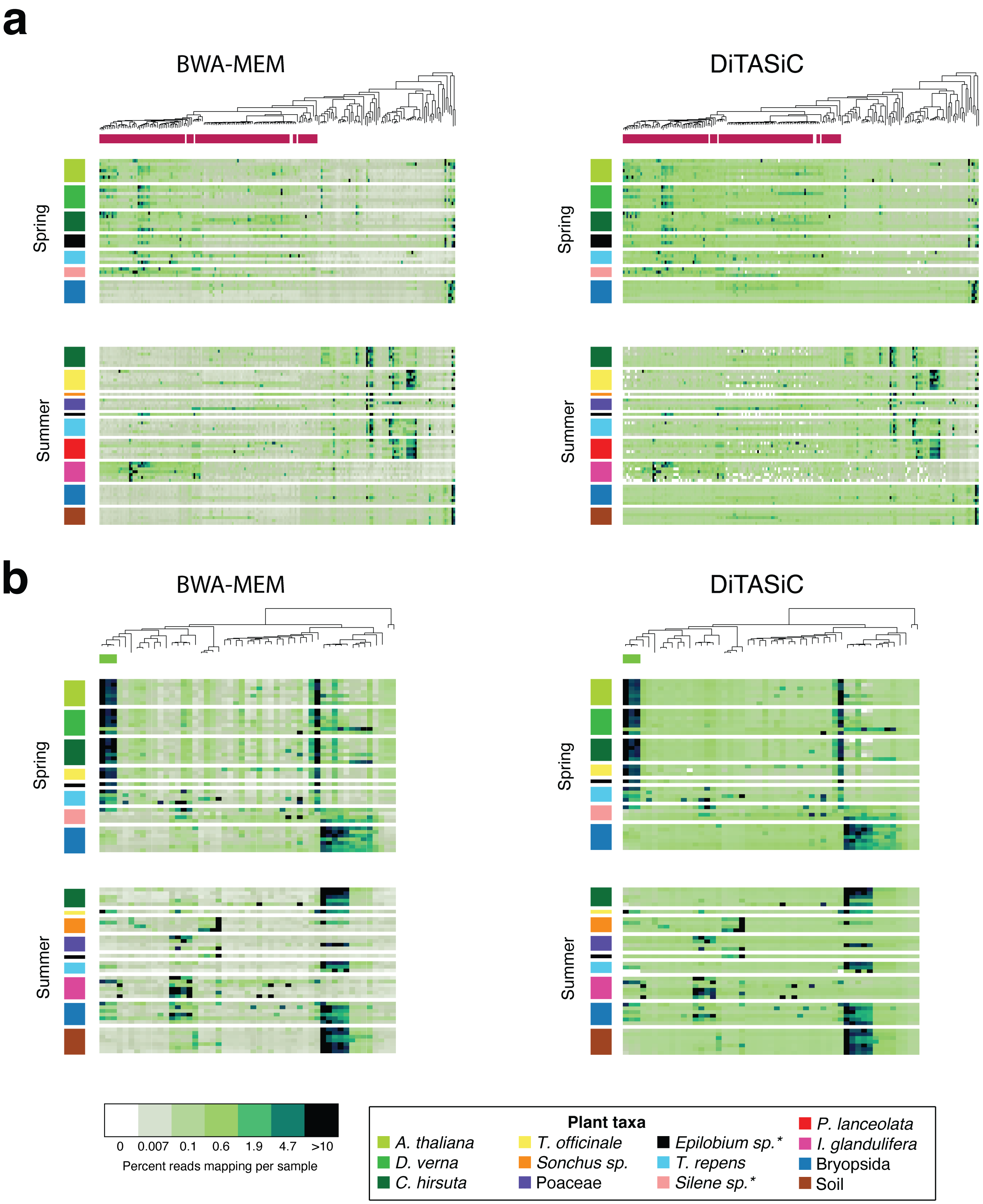

Fig. S12 | Agreement of read mapping for bulk culture metagenomes with both simple and advanced read-mapping strategies. a-b, Bulk culture metagenome reads were mapped to reference genomes either using BWA-MEM (17) as shown in Fig. 5 (left), or mapped using DiTASiC (24) (right). DiTASiC

applies a generalized linear model to resolve shared read counts. **a**, Heatmap showing fourth-root transformed reads from each *Sphingomonas* bulk culture metagenome (rows, colors from plant host taxa shown in the legend below) that map to a *Sphingomonas* local reference genome (columns) for the spring and summer collections. A * represents uncertainty in the plant taxon name. The genetic relatedness of the local bacterial reference genomes is shown by a maximum likelihood (ML) tree above the heatmap, and those reference genomes belonging to SphASV1 are indicated under the ML tree in magenta. Darker colors in the heatmap correspond to genomes attracting a greater fraction of reads in the mapping process. **b**, Same as **(a)**, but showing *Pseudomonas* bulk culture mapping to *Pseudomonas* reference genomes. Reference genomes belonging to PseASV1 are indicated under the ML tree in green.

Supporting Information References

1. X.-F. Xin, *et al.*, Bacteria establish an aqueous living space in plants crucial for virulence. *Nature* **539**, 524–529 (2016).

2. T. L. Karasov, *et al.*, Arabidopsis thaliana and Pseudomonas Pathogens Exhibit Stable Associations over Evolutionary Timescales. *Cell Host Microbe* **24**, 168–179.e4 (2018).

3. N. Rohland, D. Reich, Cost-effective, high-throughput DNA sequencing libraries for multiplexed target capture. *Genome Res.* **22**, 939–946 (2012).

4. J. Regalado, *et al.*, Combining whole-genome shotgun sequencing and rRNA gene amplicon analyses to improve detection of microbe–microbe interaction networks in plant leaves. *ISME J.* **14**, 2116–2130 (2020).

5. D. S. Lundberg, S. Yourstone, P. Mieczkowski, C. D. Jones, J. L. Dangl, Practical innovations for high-throughput amplicon sequencing. *Nat. Methods* **10**, 999–1002 (2013).

6. R. C. Edgar, Search and clustering orders of magnitude faster than BLAST. *Bioinformatics* **26**, 2460–2461 (2010).

7. J. R. Cole, *et al.*, Ribosomal Database Project: data and tools for high throughput rRNA analysis. *Nucleic Acids Res.* **42**, D633–42 (2014).

8. N. Caruccio, Preparation of next-generation sequencing libraries using Nextera™ technology: simultaneous DNA fragmentation and adaptor tagging by in vitro transposition. *Methods Mol. Biol.* **733**, 241–255 (2011).

9. A. Bankevich, *et al.*, SPAdes: a new genome assembly algorithm and its applications to single-cell sequencing. *J. Comput. Biol.* **19**, 455–477 (2012).

10. B. J. Walker, *et al.*, Pilon: an integrated tool for comprehensive microbial variant detection and genome assembly improvement. *PLoS One* **9**, e112963 (2014).

11. T. Seemann, Prokka: rapid prokaryotic genome annotation. *Bioinformatics* **30**, 2068–2069 (2014).

12. H. den Bakker, *hcdenbakker - Overview* (Github) (March 21, 2021).

13. F. A. Simão, R. M. Waterhouse, P. Ioannidis, E. V. Kriventseva, E. M. Zdobnov, BUSCO: assessing genome assembly and annotation completeness with single-copy orthologs. *Bioinformatics* **31**, 3210–3212 (2015).

14. S. Koren, A. M. Phillippy, One chromosome, one contig: complete microbial genomes from long-read sequencing and assembly. *Curr. Opin. Microbiol.* **23**, 110–120 (2015).

15. R. R. Wick, L. M. Judd, K. E. Holt, Performance of neural network basecalling tools for Oxford Nanopore sequencing. *Genome Biol.* **20**, 129 (2019).

16. H. Li, Minimap and miniasm: fast mapping and de novo assembly for noisy long sequences. *Bioinformatics* **32**, 2103–2110 (2016).

17. H. Li, Aligning sequence reads, clone sequences and assembly contigs with BWA-MEM. *arXiv [q-bio.GN]* (2013).

18. W. Ding, F. Baumdicker, R. A. Neher, panX: pan-genome analysis and exploration. *Nucleic Acids Res.* **46**, e5 (2018).

19. B. Buchfink, C. Xie, D. H. Huson, Fast and sensitive protein alignment using DIAMOND. *Nat. Methods* **12**, 59–60 (2015).

20. S. F. Altschul, W. Gish, W. Miller, E. W. Myers, D. J. Lipman, Basic local alignment search tool. *J. Mol. Biol.* **215**, 403–410 (1990).

21. M. Stanke, S. Waack, Gene prediction with a hidden Markov model and a new intron submodel. *Bioinformatics* **19 Suppl 2**, ii215–25 (2003).

22. H. Jiang, R. Lei, S.-W. Ding, S. Zhu, Skewer: a fast and accurate adapter trimmer for next-generation sequencing paired-end reads. *BMC Bioinformatics* **15**, 182 (2014).

23. H. Li, *et al.*, The Sequence Alignment/Map format and SAMtools. *Bioinformatics* **25**, 2078–2079 (2009).

24. M. Fischer, B. Strauch, B. Y. Renard, Abundance estimation and differential testing on strain level in metagenomics data. *Bioinformatics* **33**, i124–i132 (2017).

25. Y. Bai, *et al.*, Functional overlap of the Arabidopsis leaf and root microbiota. *Nature* **528**, 364–369 (2015).

26. N. A. O'Leary, *et al.*, Reference sequence (RefSeq) database at NCBI: current status, taxonomic expansion, and functional annotation. *Nucleic Acids Res.* **44**, D733–45 (2016).

27. B. D. Ondov, *et al.*, Mash: fast genome and metagenome distance estimation using MinHash. *Genome Biol.* **17**, 132 (2016).

28. C. Jain, L. M. Rodriguez-R, A. M. Phillippy, K. T. Konstantinidis, S. Aluru, High throughput ANI analysis of 90K prokaryotic genomes reveals clear species boundaries. *Nat. Commun.* **9**, 5114 (2018).

29. G. R. Warnes, *et al.*, gplots: Various R Programming Tools for Plotting Data (2020).

30. C. A. Barragan, *et al.*, RPW8/HR repeats control NLR activation in Arabidopsis thaliana. *PLoS Genet.* **15**, e1008313 (2019).

31. B. Laflamme, *et al.*, The pan-genome effector-triggered immunity landscape of a host-pathogen interaction. *Science* **367**, 763–768 (2020).

32. D. S. Lundberg, *et al.*, Host-associated microbe PCR (hamPCR): accessing new biology through convenient measurement of both microbial load and community composition. *bioRxiv*, 10.1101/2020.05.19.103937 (2020).

33. C. Vogel, N. Bodenhausen, W. Gruissem, J. A. Vorholt, The Arabidopsis leaf transcriptome reveals distinct but also overlapping responses to colonization by phyllosphere commensals and pathogen infection with impact on plant health. *New Phytol.* **212**, 192–207 (2016).

34. C. Vogel, G. Innerebner, J. Zingg, J. Guder, J. A. Vorholt, Forward genetic in planta screen for identification of plant-protective traits of Sphingomonas sp. strain Fr1 against Pseudomonas syringae DC3000. *Appl. Environ. Microbiol.* **78**, 5529–5535 (2012).

35. K. Jakob, J. M. Kniskern, J. Bergelson, The role of pectate lyase and the jasmonic acid defense response in Pseudomonas viridiflava virulence. *Mol. Plant. Microbe. Interact.* **20**, 146–158 (2007).

36. J. Ordon, *et al.*, Generation of chromosomal deletions in dicotyledonous plants employing a user-friendly genome editing toolkit. *Plant J.* **89**, 155–168 (2017).

37. E. M. Goss, J. Bergelson, Fitness consequences of infection of Arabidopsis thaliana with its natural bacterial pathogen Pseudomonas viridiflava. *Oecologia* **152**, 71–81 (2007).

38. O. Shalev, T. L. Karasov, D. S. Lundberg, H. Ashkenazy, D. Weigel, Protective host-dependent antagonism among Pseudomonas in the Arabidopsis phyllosphere. *bioRxiv*, 2021.04.08.438928 (2021).

39. Y. He, E.-H. Chung, D. A. Hubert, P. Tornero, J. L. Dangl, Specific missense alleles of the arabidopsis jasmonic acid co-receptor COI1 regulate innate immune receptor accumulation and function. *PLoS Genet.* **8**, e1003018 (2012).

40. D. S. Lundberg, *et al.*, Host-associated microbe PCR (hamPCR) enables convenient measurement of both microbial load and community composition. *Elife* **10** (2021).
